# Comparison of the efficacy of different protocols of repetitive transcranial magnetic stimulation and transcranial direct current stimulation on motor function, activities of daily living, and neurological function in patients with early stroke: a systematic review and network meta-analysis

**DOI:** 10.1007/s10072-025-08000-5

**Published:** 2025-02-05

**Authors:** Xueyi Ni, Zinan Yuan, Ruimou Xie, Xiaoxue Zhai, Xiang Cheng, Yu Pan

**Affiliations:** Department of Rehabilitation Medicine, Beijing Tsinghua Changgung Hospital, Beijing, 102218 China

**Keywords:** rTMS, tDCS, Early stroke, Motor function, ADL, Network meta-analysis

## Abstract

**Background:**

The application of transcranial direct current stimulation (tDCS) and repetitive transcranial magnetic stimulation (rTMS) in patients with early stroke has recently received considerable attention, but the optimal protocol remains inconclusive. This study intends to evaluate and compare the effects of different protocols of tDCS and rTMS on improving motor function, activities of daily living (ADL), and neurological function in patients with early stroke, and to comprehensively assess their efficacy and safety.

**Methods:**

MEDLINE, Embase, Cochrane Library, and Web of Science were searched. Risk of bias (RoB) was assessed using the Cochrane Risk of Bias 2.0 tool, and Bayesian NMA was conducted using R4.3.1 and Stata16.

**Results:**

The results of NMA showed that after early intervention, bilateral application of high- and low-frequency rTMS (BL-rTMS) performed best in improving the upper extremity motor function at the end of intervention (SUCRA: 92.8%) and 3 months (SUCRA: 95.4%). Besides, low-frequency rTMS (LF-rTMS) performed best in improving the lower extremity motor function (SUCRA: 67.7%). BL-rTMS was the most effective in ameliorating the ADL at the end of intervention (SUCRA: 100%) and 3 months (SUCRA: 85.6%). In terms of the NIHSS scores, BL-rTMS had the highest probability of being the most effective measure at the end of intervention (SUCRA: 99.7%) and 3 months (SUCRA: 97.05%). Besides, LF-rTMS (0%), 5 Hz-rTMS (0%), and intermittent theta-burst stimulation (iTBS) (0%) all exhibited a good safety profile.

**Conclusion:**

BL-rTMS is the optimal stimulation protocol for improving upper extremity motor function, ADL, and neurological function in early stroke, with long-term efficacy.

**Supplementary Information:**

The online version contains supplementary material available at 10.1007/s10072-025-08000-5.

## Introduction

Globally, stroke is the leading cause of death and disability, with approximately 795,000 new or recurrent cases of stroke annually [1]. Stroke can lead to a variety of functional impairments, such as dysphagia, cognitive impairment, dyskinesia, post-stroke pain, and depression, further affecting the activities of daily living (ADL), and resulting in a great economic cost for post-stroke care [2]. The burden of stroke is projected to rise further with an aging population and an increase in non-communicable diseases [3]. Therefore, post-stroke early rehabilitation interventions are crucial.

Early post-stroke period (acute and early subacute) is a critical period for neuroplasticity, in which the body undergoes a series of endogenous molecular, cellular, and electrophysiologic processes, thereby enhancing neurological recovery [4]. Research suggests that this process is completed almost within 30 days [5, 6]. How to intervene effectively in an early stage, a critical period with optimal brain plasticity, to enhance neuroplasticity or even prolong the remodeling duration has been one of the most important concerns of scholars [4, 7–9] Prompt and effective post-stroke rehabilitation interventions can help patients restore function and improve activity and participation abilities, which is an effective way to reduce the burden of stroke. In recent years, substantial evidence suggests that non-invasive brain stimulation (NIBS), especially transcranial direct current stimulation (tDCS) and repetitive transcranial magnetic stimulation (rTMS), is suitable for early stroke patients [10–14]. Several studies have explored NIBS as a complement to routine physiotherapy for early stroke, achieving beneficial effects [11, 15, 16]. A meta-analysis has shown that early NIBS is effective in ameliorating extremity motor function and ADL in acute stroke [17, 18]. The difference in the efficacy of different protocols of early tDCS has been compared in a study [19], and it is found that dual-tDCS is more effective in improving motor function in early stroke patients. Other studies [15, 20] compared the efficacy of high-frequency rTMS (HF-rTMS) and low-frequency rTMS (LF-rTMS), and showed that the two protocols had no significant difference in motor function improvement, but their effects were significantly better than sham.

Due to methodological limitations, the possible differences in treatment outcomes of rTMS and tDCS fail to be fully clarified. The optimal stimulation mode, long-term efficacy, and safety of early NIBS remain unclear. Network meta-analysis (NMA) can provide a more complete review of the efficacy of early tDCS and rTMS on stroke and should be considered the highest level of evidence in the treatment guidelines [21]. NMA enables simultaneous comparisons of the effects of multiple interventions, as direct comparisons among these stimulations are lacking. In addition, NMA allows for the ranking of interventions, thereby helping health professionals and clinicians to make evidence-based decisions. This study, by an NMA, intends to compare the short- and long-term effects of different protocols of tDCS and rTMS on improving motor function, ADL, and neurological function in patients with early stroke and the safety of early intervention. The findings will offer a rationale for the use of NIBS in early stroke patients and help clinical practitioners develop evidence-based treatment protocols, thereby enhancing the rehabilitation outcomes of stroke patients and reducing the social and economic burdens of stroke.

## Materials and methods

### Study protocol

This systematic review and NMA followed the PRISMA 2020 Statement [22] and PRISMA extension for network meta-analyses [23], and was registered with PROSPERO (http://www.crd.york.ac.uk/prospero/) (CRD42023492164).

### Search strategy

We searched MEDLINE, Embase, Cochrane Library, and Web of Science for randomized controlled trials (RCTs) from inception to October 7, 2023. Medical subject headings with free words were used, including “Stroke*”, “Cerebrovascular Accident*”, “Brain Vascular Accident*”, “Transcranial Direct Current Stimulation”, “Transcranial Magnetic Stimulation”, “Non-invasive Brain Stimulation*”, “Acute”, “Early Stage”, and “Randomized Controlled Trial”. The search strategy is detailed in the Supplementary File 1.

### Eligibility criteria

For this review, “early intervention” was defined as the initiation of NIBS, including different protocols of TMS and tDCS, within one month after acute stroke onset. The National Institutes of Health Stroke Scale (NIHSS) was applied to assess neurological function, Fugl-Meyer assessment scale (FMA) to assess motor function, including FMA for upper extremity (FMA-UE) and FMA for lower extremity (FMA-LE), modified Barthel index (mBI) to assess ADL, and adverse events (AEs) to assess the safety of early intervention.

We included studies that met the following criteria: (1) Population: adults ≥ 18 years; patients diagnosed with a stroke by computed tomography or magnetic resonance; stoke onset ≤ 1 month. (2) Intervention: The intervention of the experiment group included tDCS and/or rTMS. (3) Comparison: Control measures included conventional treatment and the sham or blank of rTMS/tDCS; or other types of rTMS/tDCS. (4) Outcome: At least one of the following outcomes was reported: (a) Motor function measured by FMA (FMA-UE, and FMA-LE); (b) ADL assessed by mBI; (c) safety assessed by AEs; (d) Stroke severity measured by NIHSS. (5) Study type: RCTs. (6) Language: English.

The exclusion criteria were as follows: (1) Transient ischemia attack (TIA). (2) Duration of disease > 30 days. (3) Non-RCTs such as case reports, literature reviews, conference abstracts, animal experiments, and retrospective studies. (4) Studies with incomplete data and original data unavailable after emailing the author. (5) Duplicate publications.

### Data extraction

All studies retrieved were checked for eligibility independently by two reviewers (XYN and ZNY) based on the above criteria. After duplicate publications were excluded using EndNote 21 (Clarivate Analytics, Philadelphia, PA, USA), potentially eligible studies were identified by reading titles and abstracts. Then the full text was further read to finally include eligible studies. Disagreement could be resolved by discussion with a third reviewer (YP). The following information was extracted from the included studies: first author, year of publication, sample size, age, sex, type of stroke, duration of disease, interventions, comparisons, and outcomes mentioned above.

### Quality assessment

Risk of bias (RoB) was assessed independently by two reviewers (XYN and ZNY) using the Cochrane Risk of Bias 2.0 tool [24] from the deviations from intended interventions, randomization process, measurement of the outcome, missing outcome data, selection of the reported result, and overall bias. The studies were classified as “low risk”, “high risk”, and “some concerns”. Disagreement could be resolved by discussion with a third reviewer (YP).

### Synthesis methods

R4.3.1 and Stata16 were used for calculation and plotting. Continuous outcomes (including FMA, mBI, and NIHSS) were described by mean difference (MD) and 95% confidence interval (CI). The Markov Chain Monte Carlo method was utilized, with four chains running and 2,0000 iterations of pre-simulation for annealing. The surface under the cumulative ranking curve (SUCRA, 0–100%) was calculated to estimate the probability of being the optimal intervention. Local inconsistency between direct and indirect evidence was checked by the node-splitting method. Using the Bayesian information criterion, consistency and inconsistency models were fit. The I^2^ values of 25%, 50%, and 75% corresponded to low, moderate, and high degrees of heterogeneity, respectively. Network diagrams were plotted using Stata. The publication bias in primary outcome measures was detected by funnel plots.

## Results

### Study selection

**Fig. 1:**
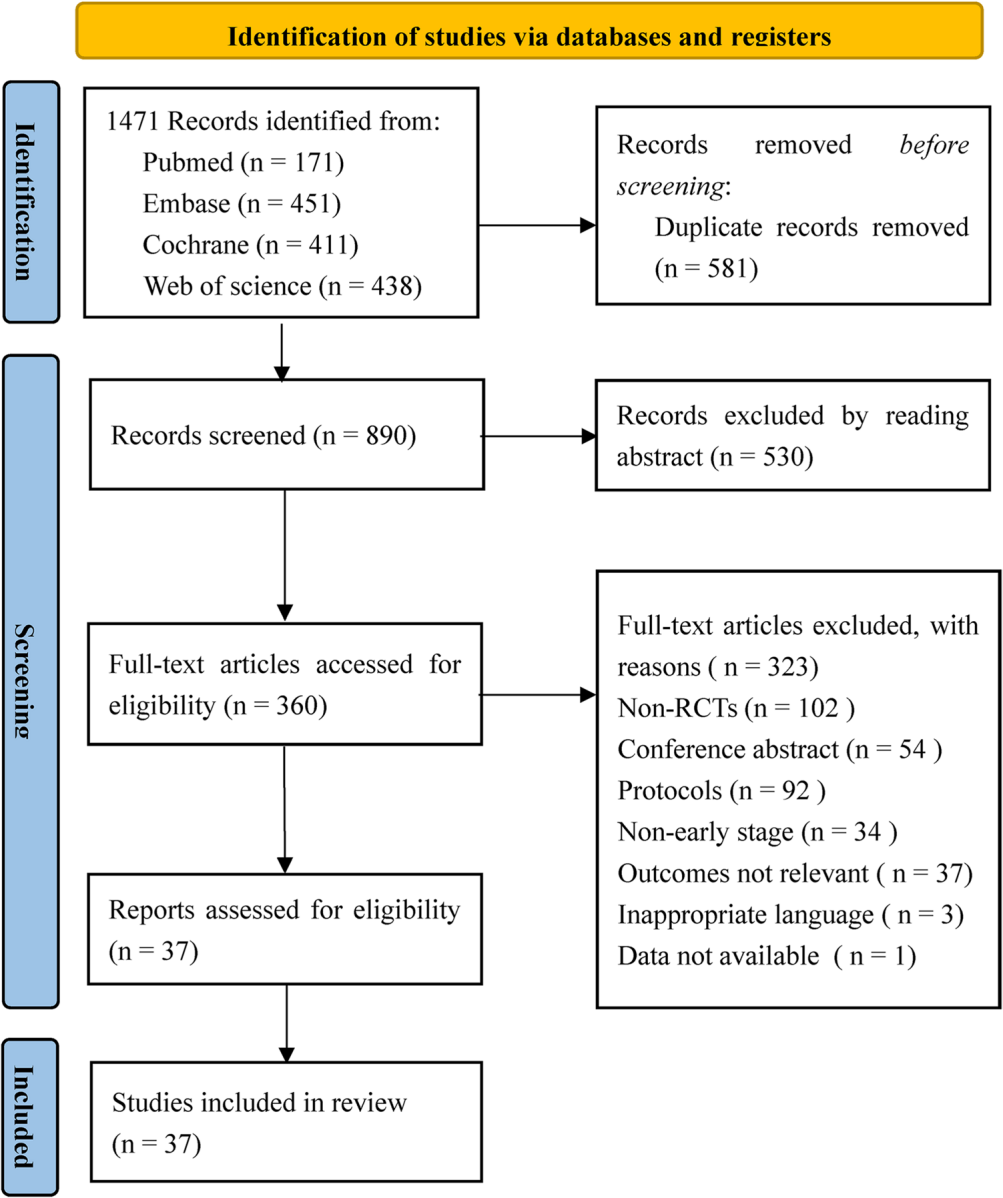
Flowchart of the study selection process

A total of 1471 studies were initially retrieved. After 581 duplicate publications were excluded, another 530 non-relevant studies were excluded by reading titles and abstracts. Among the remaining 360 studies, 102 non-RCTs, 54 conference abstracts, 92 study protocols, and 75 RCTs that did not satisfy the above eligibility criteria were excluded. Finally, 37 studies were included [11, 15, 16, 19, 20, 25–56]. The study screening process is described in Fig. 1.

### Study characteristics

A total of 1766 patients were included in the 37 studies [11, 15, 16, 19, 20, 25–56] involving 10 types of rTMS and tDCS. There were 271 cases in the HF-rTMS (≥ 10 Hz) group [15, 16, 20, 26, 30, 33, 43, 47, 51, 53, 54], 27 cases in the 5 Hz-rTMS group [39, 42], 77 cases in the 3 Hz-rTMS group [44, 53, 55, 56], 301 cases in the LF-rTMS (≤ 1 Hz) group [11, 15, 16, 20, 26, 32, 37, 38, 44, 45, 48, 51, 55], 99 cases in the bilateral application of high- and low-frequency repetitive transcranial magnetic stimulation (BL-rTMS) group [16, 31, 38, 47], 28 cases in the continuous theta-burst stimulation (cTBS) group [27], 28 cases in the intermittent theta-burst stimulation (iTBS) group [35, 37], 57 cases in the cathodal-tDCS group [11, 19, 40, 49], 83 cases in the anodal-tDCS group [19, 36, 46, 52], and 137 cases in the dual-tDCS group [19, 25, 28, 29, 34, 41, 50] The study characteristics are detailed in Table 1.

**Table 1:** Characteristics of included studies

Study	Sample size (E/C)	Age (E/C)	Gender (M/F)	Stroke type (infarction/ hemorrhage)	Onset time (d)	Intervention (E/C)	Outcome
Yagüe [25]	9/9	56 ± 14.2 / 59.4 ± 12.6	11/7	9/9	(5–17)	dual-tDCS / Sham	①
Wang [26]	80/80/80	63.85 ± 9.54 / 63.92 ± 10.28 / 64.10 ± 9.96	157/83	240/0	21.33 ± 3.06	HF-rTMS / LF-rTMS / Sham	① ③ ④ ⑥
Vink [27]	28/31	56.8 ± 12 / 63.4 ± 12	40/19	50/9	16 ± 4	cTBS / Sham	① ② ④ ⑤ ⑧
Garrido [28]	35/35	65 ± 12 / 65 ± 14	37/33	68/2	6 ± 4	dual-tDCS / Sham	① ②
Zhao [29]	30/30	59.57 ± 10.41 / 62.87 ± 8.40	39/21	60/0	7.62 ± 2.67	dual-tDCS / Control	⑧
Komatsu [30]	22/22	52(47,62) / 57(49,63)	30/14	0/44	9(6–12)	HF-rTMS / Control	⑧
Klomjai [19]	18/21/20/20	61.11 ± 9.70 / 59.94 ± 9.80 / 57.20 ± 12.54 / 60.18 ± 10.20	49/30	79/0	3.88 ± 1.34	cathodal-tDCS / anodal-tDCS / dual-tDCS / Sham	⑧
Juan [15]	15/17/14	51 ± 10 / 56 ± 10 / 52 ± 11	38/8	46/0	4.61 ± 3.45	HF-rTMS / LF-rTMS / Sham	① ②
Chen [31]	22/22	56.0(39.3,64.0) / 60.5(54.3,65.5)	33/11	44/0	6.25 ± 3.95	BL-rTMS / Sham	① ② ⑤ ⑧
Chen [16]	25/25/25/25	58.0(44.5,65.5) / 62.0(49.0,67.0) / 63.0(43.0,67.0) / 65.0(52.0,73.0)	70/30	100/0	(4–14)	BL-rTMS / HF-rTMS / LF-rTMS / Sham	① ② ④ ⑤ ⑥ ⑦ ⑧
Miu [11]	25/26	63.2 ± 12.8 / 66.5 ± 10.1	25/26	37/14	within 1 month	LF-rTMS / cathodal-tDCS	① ④
Kim [22]	8/12	67.00 ± 12.92 / 62.17 ± 16.25	12/8	20/0	within 1 month	LF-rTMS / Control	① ③ ④ ⑧
Ke [33]	32/16	55.6 ± 8.86 / 58.3 ± 8.3	20/20	48/0	11.6 ± 2.85	HF-rTMS / Sham	⑧
Bolognini [34]	16/16	68 ± 13 / 69 ± 8.5	21/11	25/7	(2–3)	dual-tDCS / Sham	④ ⑥ ⑧
Khan [35]	20/20	63.55 ± 12.67 / 64.60 ± 12.99	25/15	40/0	16.78 ± 5.12	iTBS / Control	① ② ④ ⑤ ⑥ ⑦
Du [20]	20/20/20	54 ± 12 / 56 ± 9 / 56 ± 11	48/12	60/0	5 ± 3.73	HF-rTMS / LF-rTMS / Sham	① ② ⑥ ⑧
Bornheim [36]	25/25	62.48 ± 11.86 / 63.48 ± 12.94	33/17	50/0	within 1 month	anodal-tDCS / Sham	⑧
Watanabe [37]	8/7/6	72.5 ± 6.5 / 67.6 ± 6.4 / 75.2 ± 5.5	14/7	21/0	within 7 days	iTBS / LF-rTMS / Sham	⑧
Long [38]	21/21/20	57.00 ± 11.78 / 55.90 ± 8.89 / 56.85 ± 5.48	47/15	32/30	19.49 ± 2.67	LF-rTMS / BL-rTMS / Sham	① ② ⑧
Li [39]	6/6	56.9(30–76)	1/11	12/0	within 1 week	5 Hz-rTMS / Sham	⑧
Rabadi [40]	8/8	62 ± 11 / 63 ± 6	0/16	16/0	6.4 ± 3.2	cathodal-tDCS / Sham	⑧
Oveisgharan [41]	10/10	52.1 ± 12.8 / 65.3 ± 16.5	10/10	20/0	3.0 ± 4.1	dual-tDCS / Sham	① ⑧
Guan [42]	21/21	59.7 ± 6.8 / 57.4 ± 14.0	30/12	42/0	4.6 ± 3.7	5 Hz-rTMS / Sham	① ② ③ ④ ⑤ ⑥ ⑦ ⑧
Guo [43]	7/8	67.71 ± 7.4 / 66.63 ± 9.24	7/8	15/0	4.80 ± 1.26	HF-rTMS / Sham	④ ⑥ ⑧
Du [44]	23/23/23	56.78 ± 8.47 / 56.78 ± 12.4 / 53.61 ± 13.55	45/24	69/0	(3–24)	3 Hz-rTMS / LF-rTMS / Sham	⑥ ⑤ ⑧
Matsuura [45]	10/10	72.2 ± 6.0 / 74.7 ± 12.7	11/9	20/0	9.6 ± 4.13	LF-rTMS / Sham	⑧
Chang [46]	12/12	59.9 ± 10.2 / 65.8 ± 10.6	15/9	24/0	16.3 ± 5.6	anodal-tDCS / Sham	③
Sasaki [47]	27/31	66.6 ± 9.5 / 62.7 ± 10.9	41/17	36/22	9.72 ± 3.29	HF-rTMS / BL-rTMS	⑧
Kim [48]	42/16	67.07 ± 7.67 / 65.51 ± 11.24	30/28	58/0	15.86 ± 11.06	LF-rTMS / Sham	⑧
Fusco [49]	5/6	58.36 ± 14.35	5/6	11/0	19.09 ± 8.04	cathodal-tDCS / Sham	① ④
Di [50]	17/17	63.12 ± 6.01/ 70.00 ± 4.49	22/12	34/0	3.25 ± 1.58	dual-tDCS / Sham	⑥ ⑦
Sasaki [51]	9/11/9	65.7 ± 9.1 / 68.6 ± 8.7 / 63.0 ± 9.3	20/9	13/16	17.4 ± 5.4	HF-rTMS / LF-rTMS / Sham	⑧
Rossi [52]	25/25	66.1 ± 14.3 / 70.3 ± 13.5	26/24	50/0	9.65 ± 2.59	anodal-tDCS / Sham	⑥ ⑧
Khedr [53]	16/16/16	58.25 ± 15.07 / 58.37 ± 13.96 / 58 ± 11.64	24/24	48/0	6.5 ± 3.63	3 Hz-rTMS / HF-rTMS / Sham	⑥ ⑧ ⑦
Chang [54]	18/10	56.4 ± 11.2 / 57.0 ± 14.5	17/11	28/0	13.4 ± 5.4	HF-rTMS / Sham	⑧
Khedr [55]	12/12/12	54.7 ± 9.7 / 59.0 ± 13.5 / 60.0 ± 9.5	19/17	36/0	17.1 ± 3.6	LF-rTMS / 3 Hz-rTMS / Sham	④ ⑤ ⑥ ⑦ ⑧
Khedr [56]	26/26	53.5 ± 9.5 / 52.2 ± 8.4	36/16	52/0	7.2 ± 1.44	3 Hz-rTMS / Sham	④ ⑥ ⑧

Data are expressed as the mean ± SD or Mean (range) for age and onset time; ①Fugl-Meyer assessment scale for upper extremity (FMA-UE); ②FMA-UE (3 month follw-up); ③Fugl-Meyer assessment scale for lower extremity (FMA-LE); ④modified Barthel Index (mBI); ⑤mBI (3 month follw-up); ⑥National Institute of Health Stroke Scale (NIHSS; ⑦NIHSS (3 month follw-up); ⑧adverse events (AE)

*E*; experimental group, *C;* control group, *F;* female, *M;* male, *d;* day, *HF-rTMS;* high-frequency repetitive transcranial magnetic stimulation, *LF-rTMS;* low-frequency repetitive transcranial magnetic stimulation, *BL-rTMS;* bilateral application of HF- rTMS and LF-rTMS, *iTBS;* intermittent theta-burst stimulation, *cTBS;* continuous theta-burst stimulation, *tDCS;* transcranial direct current stimulation

### RoB assessment

**Fig. 2:**
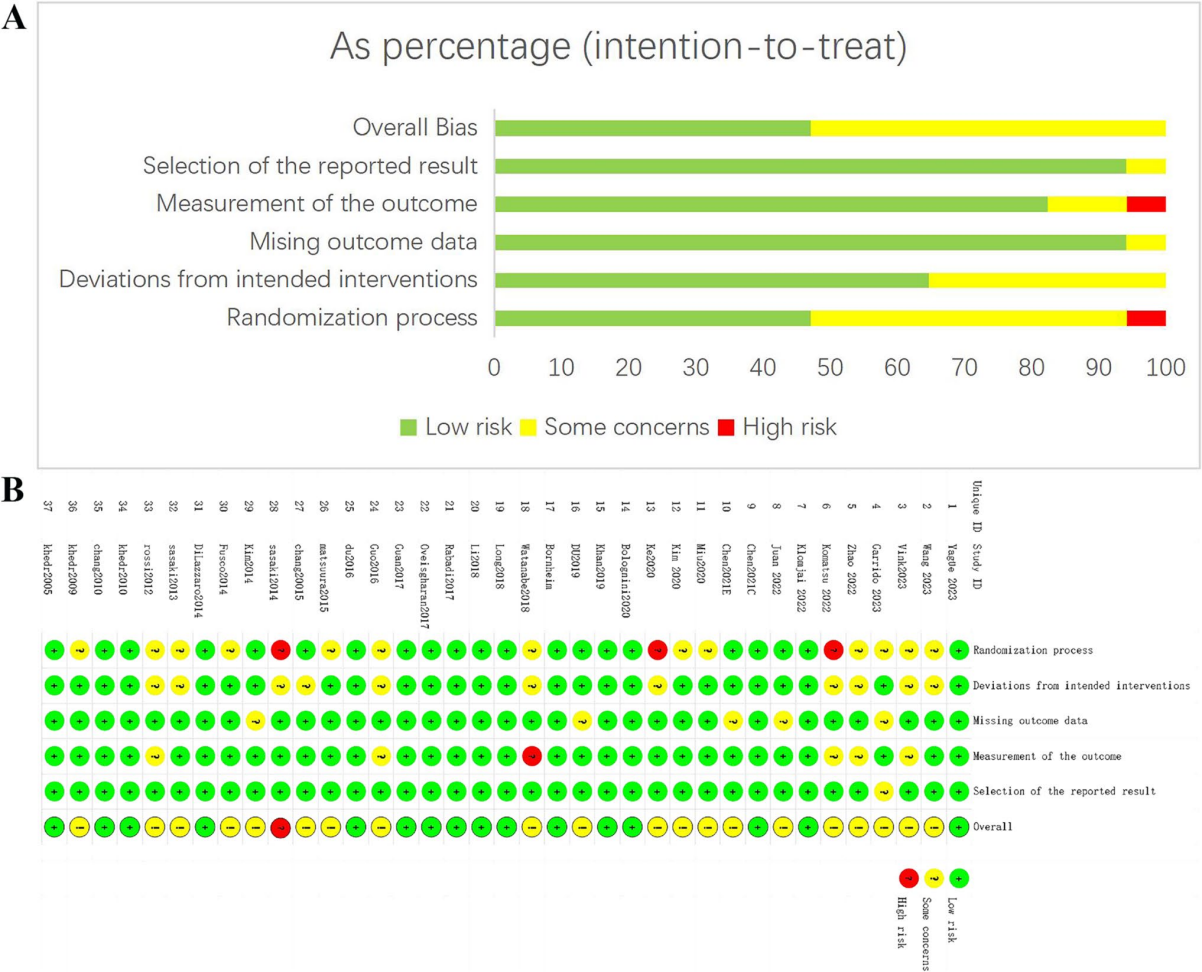
Quality assessment of selected studies by the Cochrane Risk of Bias Tool. **A** Risk of bias graph: review authors’ judgments about each risk of bias item presents as percentages across all included studies; **B** Risk of bias summary: review authors’ judgments about each risk of bias item for each included study

In terms of the randomization process, 21 studies [15, 16, 19, 20, 25, 31, 34–36, 38–42, 44, 46, 48, 50, 53, 54, 56] were rated as low risk, 13 studies [11, 26–29, 32, 37, 43, 45, 49, 51, 52, 55] as some concerns, and three studies [30, 33, 47] as high risk. In terms of deviations from intended interventions, 26 studies [11, 15, 16, 19, 20, 25, 28, 31, 32, 34–36, 38–42, 44, 45, 48–50, 53–56] were rated as low risk, and 11 studies [26, 27, 29, 30, 33, 37, 43, 46, 47, 51, 52] as some concerns. For missing outcome data, 32 [11, 19, 25–27, 29–47, 49–56] studies and 5 studies [15, 16, 20, 28, 48]were rated as low risk and some concerns, respectively. For measurement of the outcome, 31 studies [11, 15, 16, 19, 20, 25, 26, 28, 31–36, 38–42, 44–51, 53–56], five studies [27, 29, 30, 43, 52], and one study [37] were rated as low risk, some concerns, and high risk, respectively. For selection of the reported result, 36 studies [11, 15, 16, 19, 20, 25–27, 29–56] were rated as low risk, and one study [28] as some concerns. The RoB assessment results are displayed in Fig. 2. To sum up, low risk, some concerns, and high risk were identified in 16 studies [19, 25, 31, 34–36, 38–42, 44, 50, 53, 54, 56], 20 studies [11, 15, 16, 20, 26–30, 32, 33, 37, 43, 45, 46, 48, 49, 51, 52, 55], and one study [47], respectively.

### FMA

#### FMA-UE

**Fig. 3:**
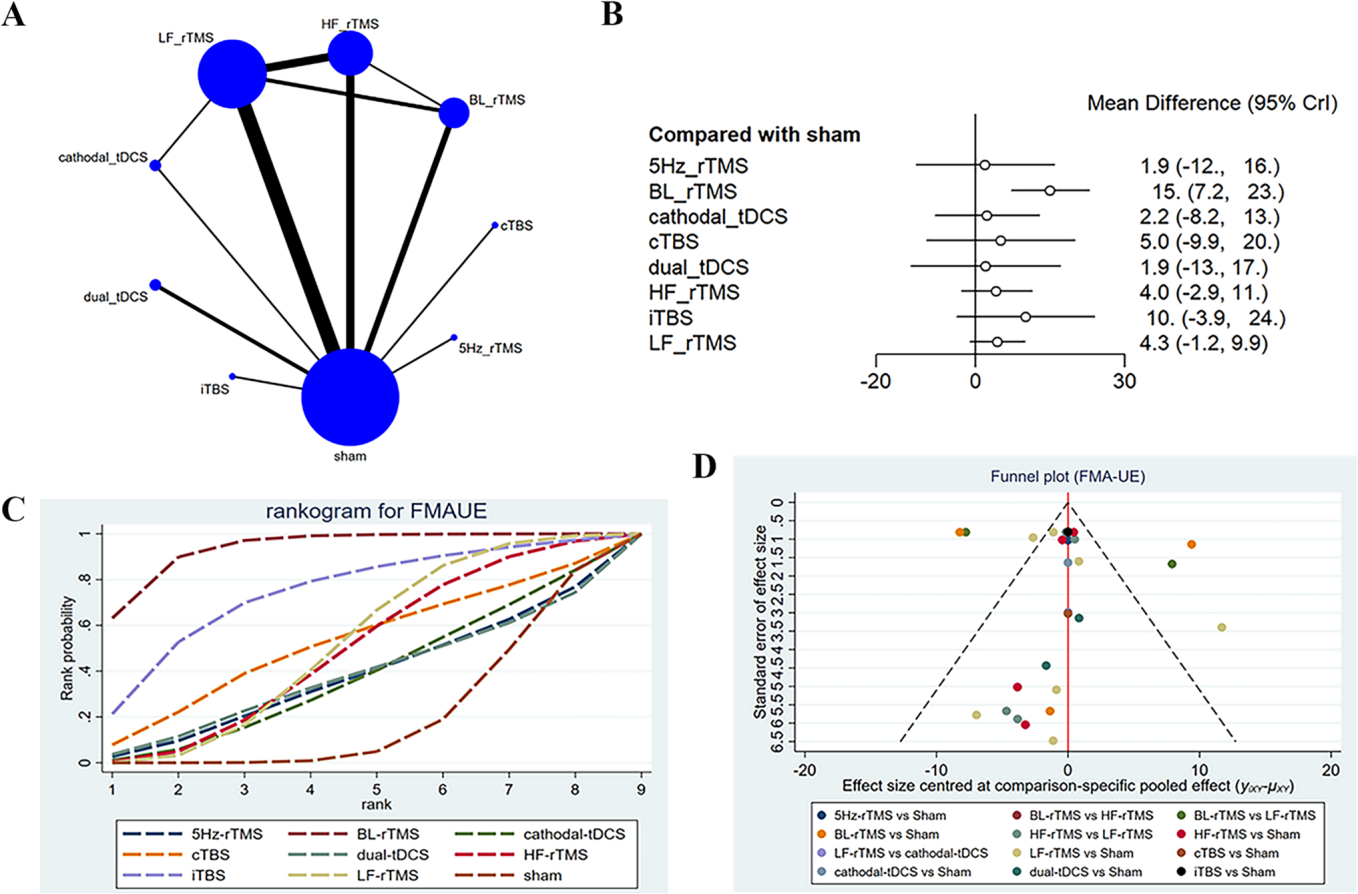
Network meta-analysis results for FMA-UE (end of treatment). **A**: Network plot; **B**: Forest plot; **C**: Cumulative probability ranking curve of different interventions; **D**: Funnel plot. HF-rTMS, high-frequency repetitive transcranial magnetic stimulation; LF-rTMS, low-frequency repetitive transcranial magnetic stimulation; BL-rTMS, bilateral application of HF- rTMS and LF-rTMS; cTBS, continuous theta-burst stimulation; iTBS, intermittent theta-burst stimulation; tDCS, transcranial direct current stimulation

FMA-UE of post-early intervention was reported in 14 studies [11, 15, 16, 20, 26–28, 31, 32, 35, 38, 41, 42, 49], with 896 patients treated with LF-rTMS, 5 Hz-rTMS, HF-rTMS, BL-rTMS, cTBS, iTBS, cathodal-tDCS, and dual-tDCS. In the network diagram (Fig. 3A**)**, the dots represented the interventions, their size represented the sample size, and the thickness of connecting lines represented the number of studies. It was found that direct comparisons were present between LF-rTMS and HF-rTMS, BL-rTMS, HF-rTMS, and cathodal-tDCS, while indirect comparisons were present between dual-tDCS, iTBS, cTBS, and 5 Hz-rTMS and other interventions. The analysis results converged satisfactorily (Supplementary File 2), and good consistency was exposed by the node-splitting method. As shown in the league table (Table 2), the efficacy of BL-rTMS was superior to cathodal-tDCS (SMD = 12.77, 95% CI: 0.02–25.41), HF-rTMS (SMD = 10.95, 95% CI: 1.43–20.11), LF-rTMS (SMD = 10.69, 95% CI: 2.45–18.97), and sham (SMD = 14.96, 95% CI: 7.18–22.84). The forest plot revealed that only BL-rTMS (SMD = 14.96, 95% CI: 7.18–22.84) was more efficacious than sham in improving FMA-UE scores (Fig. 3B). In addition, BL-rTMS had the highest probability of being effective (SUCRA: 92.8%), and acted as the optimal stimulation protocol for improving FMA-UE scores, followed by iTBS (SUCRA: 79.9%) and LF-rTMS (SUCRA: 57.0%) (Fig. 3C). The funnel plot was largely symmetric, with a few studies located outside of the funnel plot, suggesting low risk of publication bias (Fig. 3D).

**Table 2:**
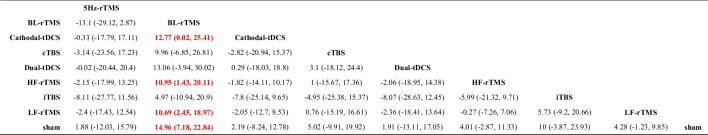
The result of network meta-analysis comparing the effect of all interventions on Fugl-Meyer assessment-upper limb (end of treatment) including SMD and 95% CI

Red and bold numbers are statistically significant. *HF-rTMS;* high-frequency repetitive transcranial magnetic stimulation, *LF-rTMS;* low-frequency repetitive transcranial magnetic stimulation, *BL-rTMS;* bilateral application of HF- rTMS and LF-rTMS, *iTBS;* intermittent theta-burst stimulation, *cTBS;* continuous theta-burst stimulation, *tDCS;* transcranial direct current stimulation

**Fig. 4:**
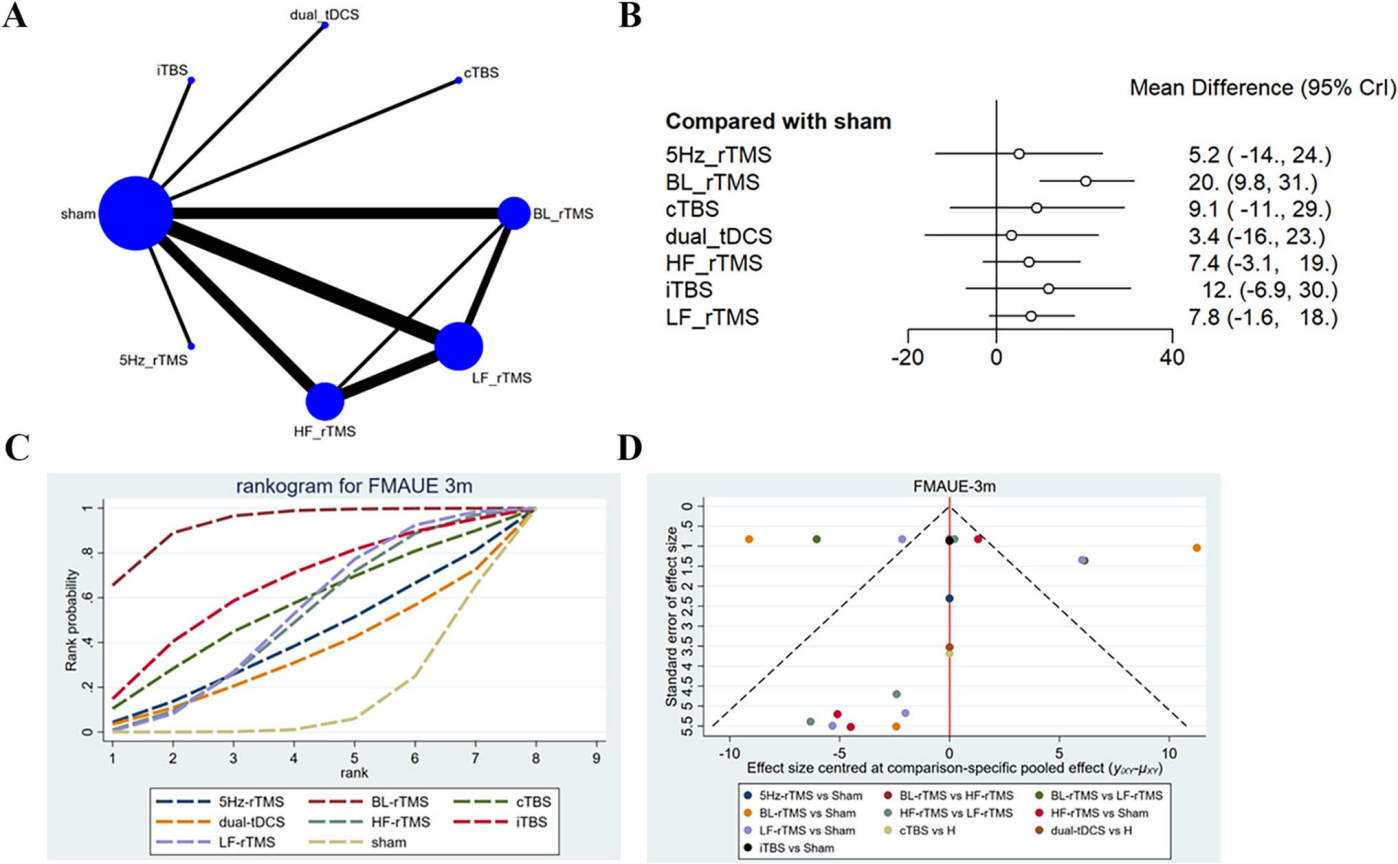
Network meta-analysis results for FMA-UE (3 month follw-up). **A**: Network plot; **B**: Forest plot; **C**: Cumulative probability ranking curve of different interventions; **D**: Funnel plot. HF-rTMS, high-frequency repetitive transcranial magnetic stimulation; LF-rTMS, low-frequency repetitive transcranial magnetic stimulation; BL-rTMS, bilateral application of HF- rTMS and LF-rTMS; cTBS, continuous theta-burst stimulation; iTBS, intermittent theta-burst stimulation; tDCS, transcranial direct current stimulation

The long-term effect (3-month follow-up) on FMA-UE was reported in nine studies [15, 16, 20, 27, 28, 31, 35, 38, 42], with 458 patients treated with LF-rTMS, 5 Hz-rTMS, HF-rTMS, BL-rTMS, cTBS, iTBS, and dual-tDCS. The network diagram showed that direct comparisons were present between LF-rTMS and HF-rTMS, BL-rTMS, and HF-rTMS, while indirect comparisons were present between dual-tDCS, iTBS, cTBS, and 5 Hz-rTMS and other interventions (Fig. 4A). The 3-month follow-up results converged satisfactorily (Supplementary File 2), and good consistency was exposed by the node-splitting method. As shown in the league table (Table 3), the efficacy of BL-rTMS was superior to LF-rTMS (SMD = 12.42, 95% CI: 0.76–23.89) and sham (SMD = 20.24, 95% CI: 9.8–31.11). The forest plot revealed that only BL-rTMS (SMD = 20.24, 95% CI: 9.8–31.11) was more effective than sham in improving FMA-UE scores (Fig. 4B). In addition, BL-rTMS had the highest probability of being effective (SUCRA: 95.4%), and acted as the optimal stimulation protocol for improving FMA-UE scores, followed by iTBS (SUCRA: 66.5%) and cTBS (SUCRA: 55.1%) (Fig. 4C). The funnel plot was largely symmetric, with some studies deviating considerably from the funnel plot, especially on the left side, suggesting that the efficacy might be underestimated in some studies (Fig. 4D**)**.

**Table 3:**
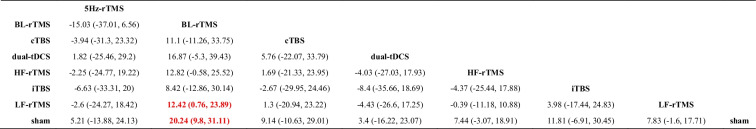
The result of network meta-analysis comparing the effect of all interventions on Fugl-Meyer assessment-upper limb (3 month follw-up) including SMD and 95% CI

Red and bold numbers are statistically significant. *HF-rTMS;* high-frequency repetitive transcranial magnetic stimulation, *LF-rTMS;* low-frequency repetitive transcranial magnetic stimulation, *BL-rTMS;* bilateral application of HF- rTMS and LF-rTMS, *iTBS;* intermittent theta-burst stimulation, *cTBS;* continuous theta-burst stimulation, *tDCS;* transcranial direct current stimulation

#### FMA-LE

**Fig. 5:**
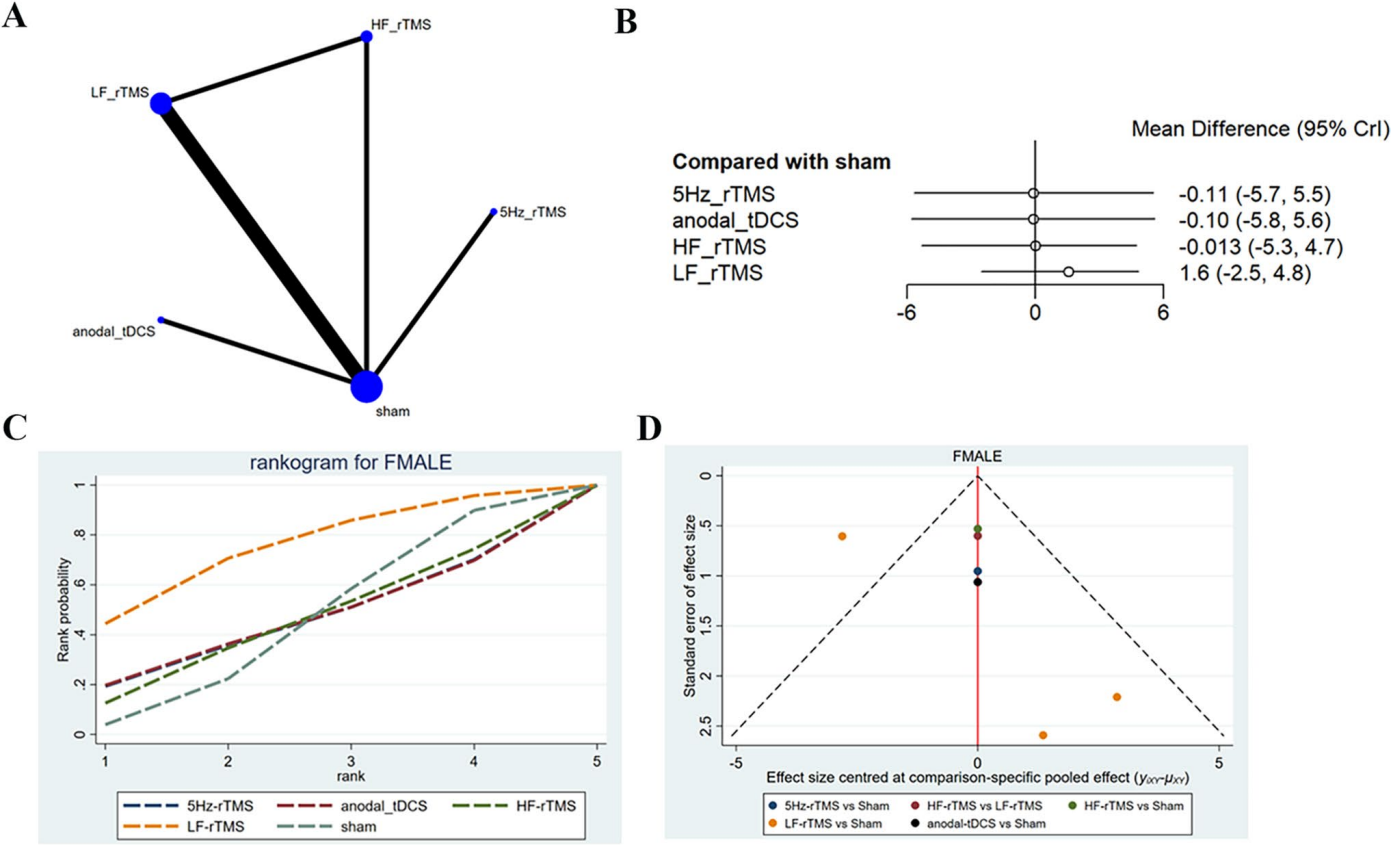
Network meta-analysis results for FMA-LE (end of treatment). **A**: Network plot; **B**: Forest plot; **C**: Cumulative probability ranking curve of different interventions; **D**: Funnel plot. HF-rTMS, high-frequency repetitive transcranial magnetic stimulation; LF-rTMS, low-frequency repetitive transcranial magnetic stimulation; tDCS, transcranial direct current stimulation

FMA-LE of post-early intervention was reported in four studies [26, 32, 42, 46], with 333 patients treated with LF-rTMS, 5 Hz-rTMS, HF-rTMS, and anodal-tDCS. The network diagram showed that LF-rTMS and HF-rTMS had direct comparisons, while anodal-tDCS and 5 Hz-rTMS had indirect comparisons with other interventions (Fig. 5A). The analysis results converged satisfactorily (Supplementary File 2). No statistically significant difference was found in the improvement of FMA-LE scores among interventions (Table 4). LF-rTMS had the highest probability of being effective (SUCRA: 67.7%), followed by 5 Hz-rTMS (SUCRA: 46.9%) and sham (SUCRA: 46.7%) (Fig. 5C**)**. The funnel plot was globally symmetric, suggesting no significant publication bias (Fig. 5D**)**.

**Table 4:** The result of network meta-analysis comparing the effect of all interventions on Fugl-Meyer assessment-lower limb (end of treatment) including SMD and 95% CI

	5 Hz-rTMS				
anodal-tDCS	0 (−7.97, 8.07)	anodal-tDCS			
HF-rTMS	−0.09 (−7.31, 7.78)	−0.08 (−7.37, 7.81)	HF-rTMS		
LF-rTMS	−1.74 (−7.9, 5.53)	−1.73 (−7.98, 5.57)	−1.7 (−6.49, 3.67)	LF-rTMS	
sham	−0.11 (−5.68, 5.52)	−0.1 (−5.8, 5.6)	−0.01 (−5.35, 4.72)	1.57 (−2.54, 4.82)	sham

HF-rTMS, high-frequency repetitive transcranial magnetic stimulation; LF-rTMS, low-frequency repetitive transcranial magnetic stimulation; tDCS, transcranial direct current stimulation

### mBI

**Fig. 6:**
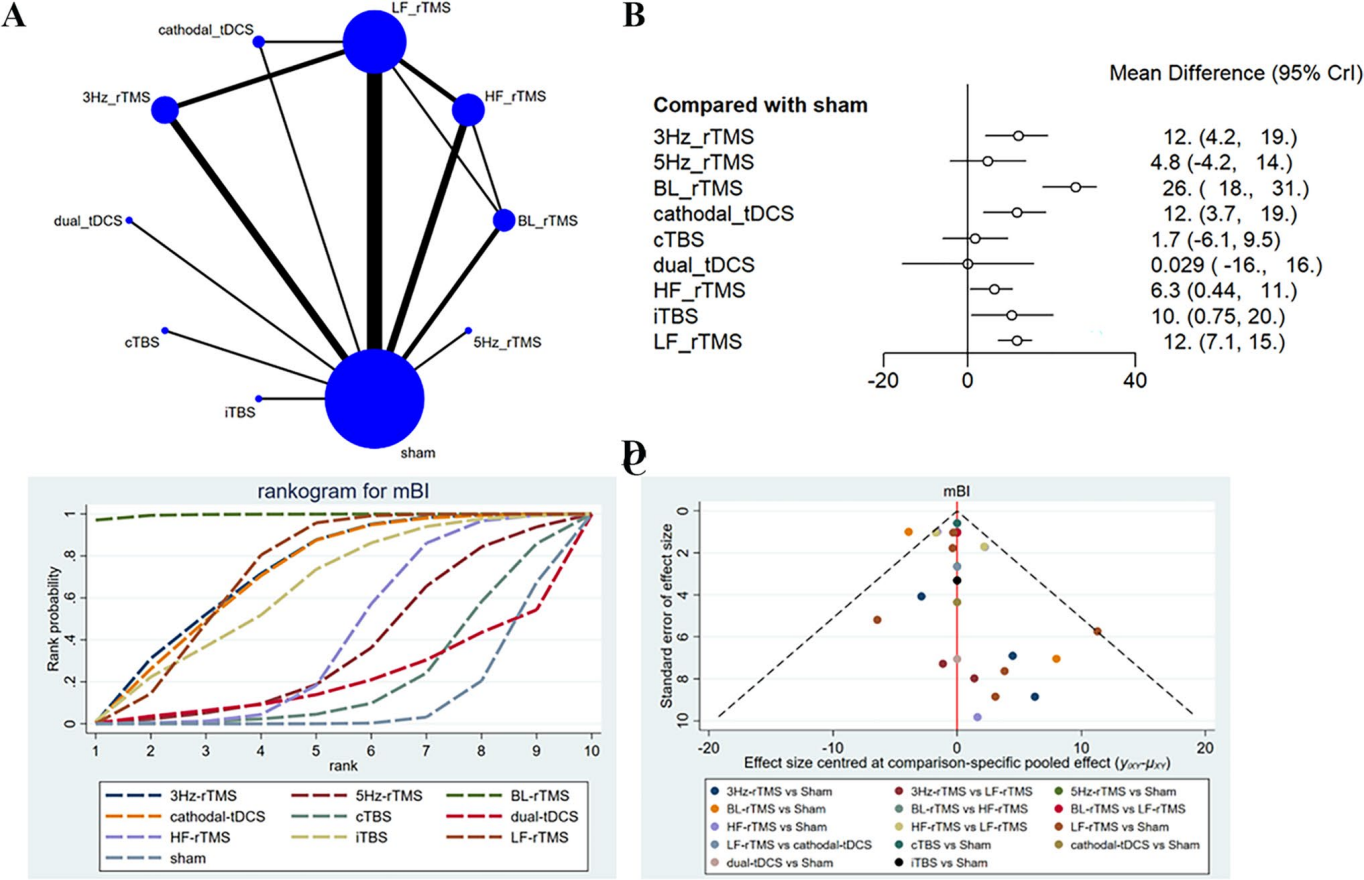
Network meta-analysis results for mBI (end of treatment). **A**: Network plot; **B**: Forest plot; **C**: Cumulative probability ranking curve of different interventions; **D**: Funnel plot. HF-rTMS, high-frequency repetitive transcranial magnetic stimulation; LF-rTMS, low-frequency repetitive transcranial magnetic stimulation; BL-rTMS, bilateral application of HF- rTMS and LF-rTMS; cTBS, continuous theta-burst stimulation; iTBS, intermittent theta-burst stimulation; tDCS, transcranial direct current stimulation

A total of 14 studies [11, 16, 26, 27, 31, 32, 34, 35, 42–44, 49, 55, 56] reported mBI, with 849 patients treated with LF-rTMS, 3 Hz-rTMS, 5 Hz-rTMS, HF-rTMS, BL-rTMS, cTBS, iTBS, cathodal-tDCS, and dual-tDCS. The network diagram showed direct comparisons of LF-rTMS with HF-rTMS, BL-rTMS, cathodal-tDCS, and 3 Hz-rTMS, and indirect comparisons of dual-tDCS, cTBS, iTBS, and 5 Hz-rTMS with other interventions (Fig. 6A**)**. The analysis results converged satisfactorily (Supplementary File 2), and good consistency was exposed by the node-splitting method. The efficacy of LF-rTMS (SMD = 11.76, 95% CI: 7.08–15.3), 3 Hz-rTMS (SMD = 11.99, 95% CI: 4.16–18.98), HF-rTMS (SMD = 6.34, 95% CI: 0.44–10.66), BL-rTMS (SMD = 25.7, 95% CI: 17.81–30.59), iTBS (SMD = 10.44, 95% CI: 0.75–20.27), and cathodal-tDCS (SMD = 11.74, 95% CI: 3.7–18.62) was better than sham (Fig. 6B**)**. As shown in the league table (Table 5), different interventions had significant differences in improving FMA-UE scores. The efficacy of BL-rTMS was better than cathodal-tDCS (SMD = 13.81, 95% CI: 4.01–22.46), cTBS (SMD = 24.09, 95% CI: 12.35–32.41), dual-tDCS (SMD = 25.4, 95% CI: 8.02–41.88), HF-rTMS (SMD = 19.37, 95% CI: 11.72–25.45), iTBS (SMD = 15.41, 95% CI: 2.11–25.61), and LF-rTMS (SMD = 13.92, 95% CI: 6.53–19.55), and LF-rTMS was also superior to cTBS (SMD=−10.16, 95% CI: −18.18 to −0.95). BL-rTMS had the highest probability of being effective (SUCRA: 100%), and acted as the optimal stimulation protocol for ameliorating FMA-UE scores, followed by 3 Hz-rTMS (SUCRA: 72.4%) and LF-rTMS (SUCRA: 72.0%) (Fig. 6C). The funnel plot was globally symmetric, suggesting no significant publication bias (Fig. 6D**)**.

**Table 5:**
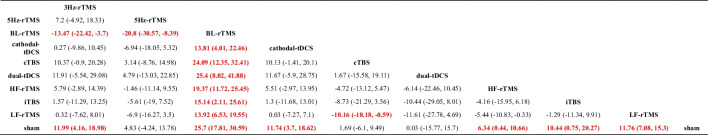
The result of network meta-analysis comparing the effect of all interventions on Modified Barthel Index (end of treatment) including SMD and 95% CI

Red and bold numbers are statistically significant. *HF-rTMS;* high-frequency repetitive transcranial magnetic stimulation, *LF-rTMS;* low-frequency repetitive transcranial magnetic stimulation, *BL-rTMS*, bilateral application of HF- rTMS and LF-rTMS, *iTBS;* intermittent theta-burst stimulation, *cTBS;* continuous theta-burst stimulation, *tDCS;* transcranial direct current stimulation

**Fig. 7:**
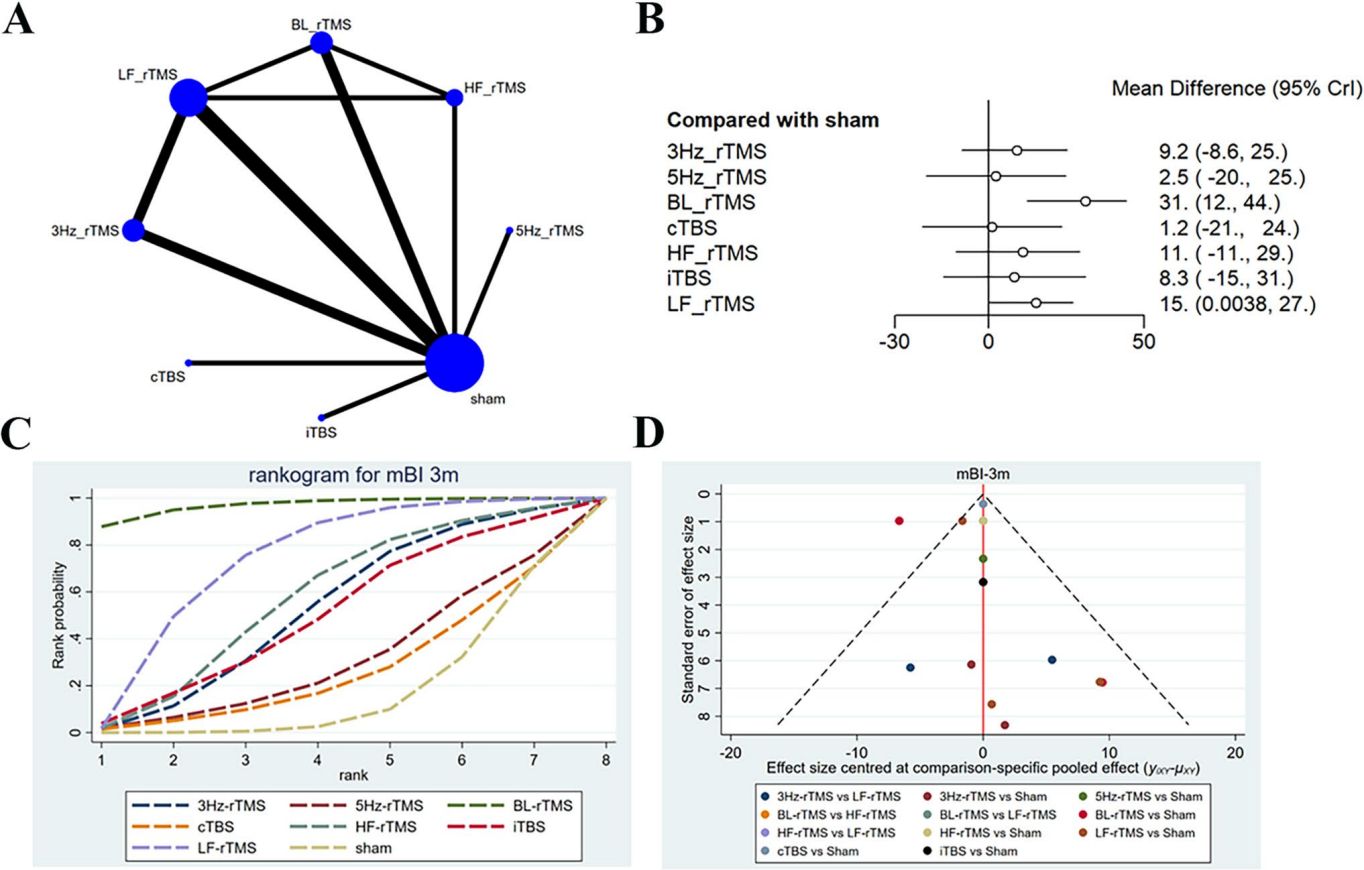
Network meta-analysis results for mBI (3 month follw-up) (**A**): Network plot; **B**: Forest plot; **C**: Cumulative probability ranking curve of different interventions; **D**: Funnel plot. HF-rTMS, high-frequency repetitive transcranial magnetic stimulation; LF-rTMS, low-frequency repetitive transcranial magnetic stimulation; BL-rTMS, bilateral application of HF- rTMS and LF-rTMS; cTBS, continuous theta-burst stimulation; iTBS, intermittent theta-burst stimulation; tDCS, transcranial direct current stimulation

Seven studies [16, 27, 31, 35, 42, 44, 55] reported the long-term effects on mBI (3-month follow-up), with 361 patients treated with LF-rTMS, 3 Hz-rTMS, 5 Hz-rTMS, HF-rTMS, BL-rTMS, cTBS, and iTBS. The network diagram showed direct comparisons of LF-rTMS with HF-rTMS, BL-rTMS, and 3 Hz-rTMS, and indirect comparisons of cTBS, iTBS, and 5 Hz-rTMS with other interventions (Fig. 7A**)**. The 3-month follow-up results converged satisfactorily (Supplementary File 2), and good consistency was exposed by the node-splitting method. The forest plot revealed that only LF-rTMS (SMD = 15.25, 95% CI: 0–27.11) and BL-rTMS (SMD = 31.15, 95% CI: 12.41–44.26) were superior to sham in the efficacy (Fig. 7B). No statistically significant difference was observed in the long-term effects of other interventions on mBI (Table 6.). BL-rTMS had the highest probability of being effective (SUCRA: 85.6%), and acted as the optimal stimulation protocol for ameliorating FMA-UE scores, followed by LF-rTMS (SUCRA: 62.2%) and 3 Hz-rTMS (SUCRA: 52.7%) (Fig. 7C**)**. The funnel plot was globally symmetric, with a few studies deviating from the funnel plot, suggesting low publication bias and reliable results (Fig. 7D**)**.

**Table 6:**
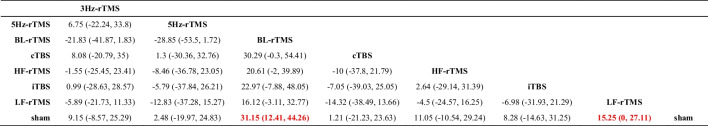
The result of network meta-analysis comparing the effect of all interventions on Modified Barthel Index limb (3 month follw-up) including SMD and 95% CI

Red and bold numbers are statistically significant. *HF-rTMS*, high-frequency repetitive transcranial magnetic stimulation, *LF-rTMS;* low-frequency repetitive transcranial magnetic stimulation, *BL-rTMS;* bilateral application of HF- rTMS and LF-rTMS, *iTBS;* intermittent theta-burst stimulation, *cTBS;* continuous theta-burst stimulation

### NIHSS

**Fig. 8:**
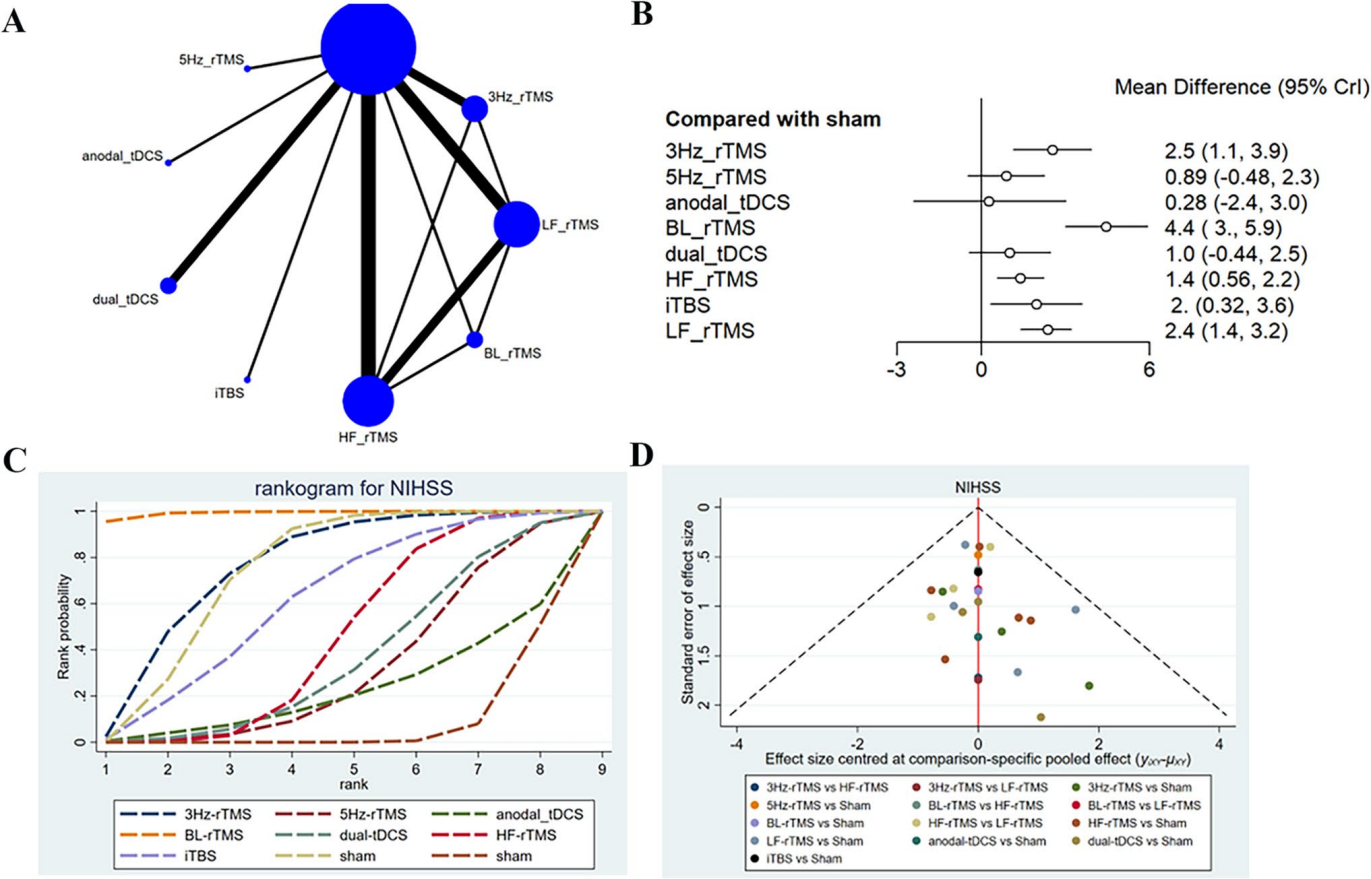
Network meta-analysis results for NIHSS (end of treatment). **A**: Network plot; **B**: Forest plot; **C**: Cumulative probability ranking curve of different interventions; **D**: Funnel plot. HF-rTMS, high-frequency repetitive transcranial magnetic stimulation; LF-rTMS, low-frequency repetitive transcranial magnetic stimulation; BL-rTMS, bilateral application of HF- rTMS and LF-rTMS; iTBS, intermittent theta-burst stimulation; tDCS, transcranial direct current stimulation

NIHSS scores of post-early intervention were described in 12 studies [16, 20, 26, 34, 35, 42, 43, 50, 52, 53, 55, 56], with 727 patients treated with LF-rTMS, 3 Hz-rTMS, 5 Hz-rTMS, HF-rTMS, BL-rTMS, iTBS, anodal-tDCS, and dual-tDCS. The network diagram revealed direct comparisons of LF-rTMS with HF-rTMS, BL-rTMS, and 3 Hz-rTMS, and indirect comparisons of dual-tDCS, iTBS, 5 Hz-rTMS, and anodal-tDCS with other interventions (Fig. 8A). The analysis results converged satisfactorily (Supplementary File 2), and good consistency was exposed by the node-splitting method. The forest plot revealed that LF-rTMS (SMD = 2.36, 95% CI: 1.4–3.19), 3 Hz-rTMS (SMD = 2.54, 95% CI: 1.13–3.91), HF-rTMS (SMD = 1.39, 95% CI: 0.56–3.58), BL-rTMS (SMD = 4.44, 95% CI: 2.98–5.91), and iTBS (SMD = 1.96, 95% CI: 0.32–20.27) outperformed sham in the efficacy (Fig. 8B). Different interventions had significant differences in the effect on NIHSS scores (Table 7). The efficacy of BL-rTMS was superior to 5 Hz-rTMS (SMD = 3.56, 95% CI: 1.54–5.56), anodal-tDCS (SMD = 4.15, 95% CI: 1.06–7.23), dual-tDCS (SMD = 3.42, 95% CI: 1.39–5.52), HF-rTMS (SMD = 3.05, 95% CI: 1.64–4.48), iTBS (SMD = 2.49, 95% CI: 0.32–4.68), and LF-rTMS (SMD = 2.09, 95% CI: 0.63–3.64). BL-rTMS had the highest probability of being effective (SUCRA: 99.7%) and acted as the optimal stimulation protocol for ameliorating FMA-UE scores, followed by 3 Hz-rTMS (SUCRA: 77.9%) and LF-rTMS (SUCRA: 75.7%) (Fig. 8C). The funnel plot was roughly symmetric, suggesting almost no publication bias, and reliable and consistent results (Fig. 8D).

**Table 7:**
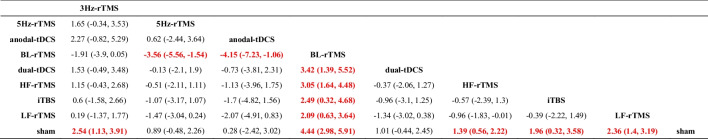
The result of network meta-analysis comparing the effect of all interventions on National Institutes of Health Stroke Scale (end of treatment) including SMD and 95% CI

Red and bold numbers are statistically significant. HF-rTMS, high-frequency repetitive transcranial magnetic stimulation; LF-rTMS, low-frequency repetitive transcranial magnetic stimulation; BL-rTMS, bilateral application of HF- rTMS and LF-rTMS; iTBS, intermittent theta-burst stimulation; tDCS, transcranial direct current stimulation

**Fig. 9:**
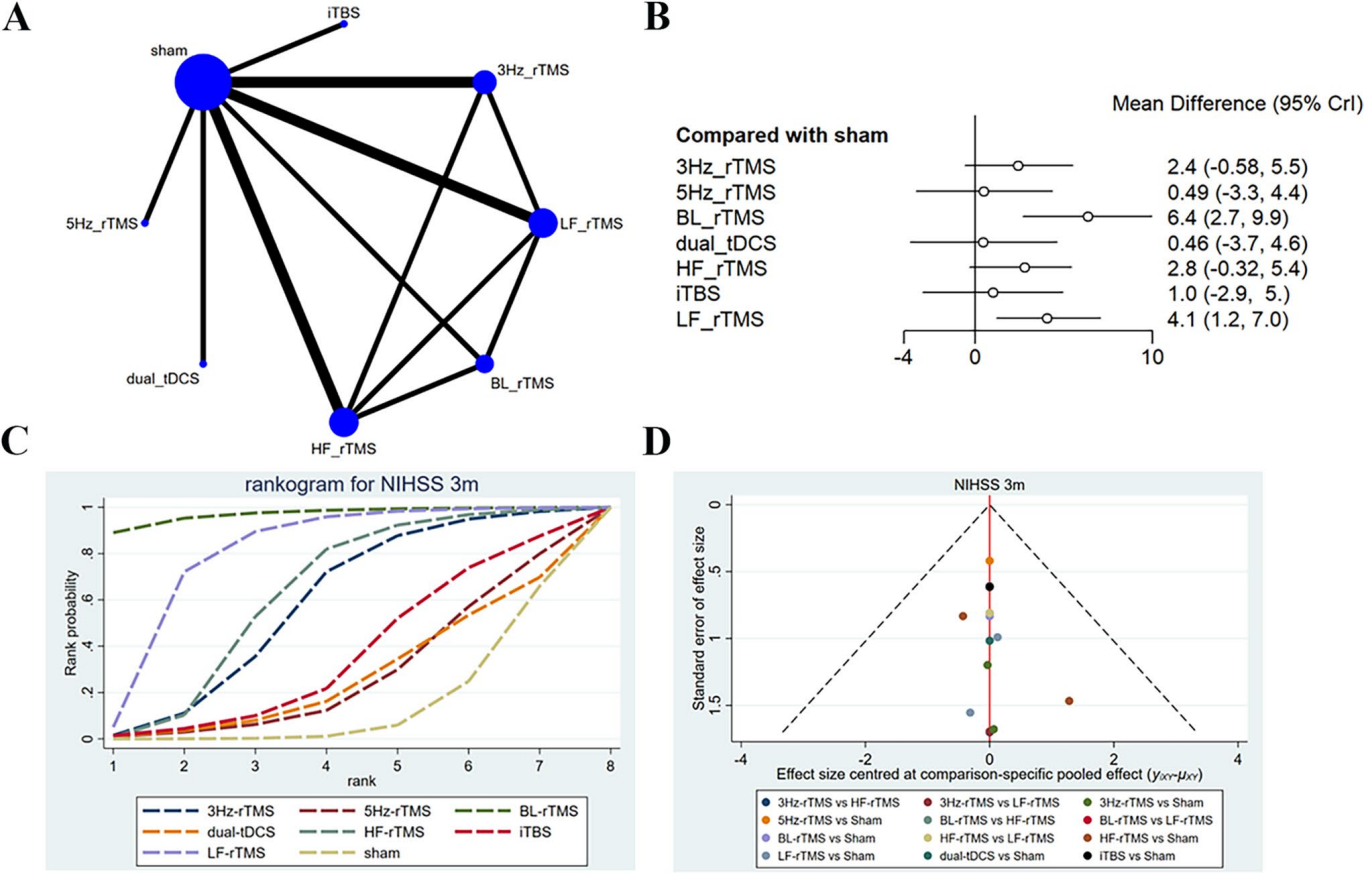
Network meta-analysis results for NIHSS (3 month follw-up). **A**: Network plot; **B**: Forest plot; **C**: Cumulative probability ranking curve of different interventions; **D**: Funnel plot. HF-rTMS, high-frequency repetitive transcranial magnetic stimulation; LF-rTMS, low-frequency repetitive transcranial magnetic stimulation; BL-rTMS, bilateral application of HF- rTMS and LF-rTMS; iTBS, intermittent theta-burst stimulation; tDCS, transcranial direct current stimulation

The long-term effects (3-month follow-up) on NIHSS scores were described in six studies [16, 35, 42, 50, 53, 55], with 271 patients treated with LF-rTMS, 3 Hz-rTMS, 5 Hz-rTMS, HF-rTMS, BL-rTMS, iTBS, and dual-tDCS. The network diagram revealed direct comparisons of LF-rTMS with HF-rTMS, BL-rTMS, and 3 Hz-rTMS, and indirect comparisons of dual-tDCS, iTBS, and 5 Hz-rTMS with other interventions (Fig. 9A). The 3-month follow-up results converged satisfactorily (Supplementary File 2), and good consistency was exposed by the node-splitting method. The forest plot revealed that only BL-rTMS (SMD = 6.36, 95% CI: 2.68–9.93) and LF-rTMS (SMD = 4.07, 95% CI: 1.21–7.04) were superior to sham in the efficacy (Fig. 9B). Different interventions had significant differences in the effect on NIHSS scores (Table 8). The efficacy of BL-rTMS was better than 5 Hz-rTMS (SMD = 5.86, 95% CI: 0.54–11.08), dual-tDCS (SMD = 4.07, 95% CI: 1.21–7.04), and HF-rTMS (SMD = 4.07, 95% CI: 1.21–7.04). BL-rTMS had the highest probability of being effective (SUCRA: 97.05%) and acted as the optimal stimulation protocol for ameliorating FMA-UE scores, followed by LF-rTMS (SUCRA: 80.0%) and HF-rTMS (SUCRA: 62.0%) (Fig. 9C). The funnel plot was largely symmetric, suggesting almost no publication bias (Fig. 9D).

**Table 8:**
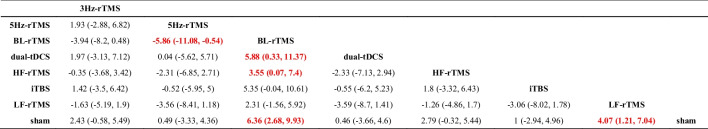
The result of network meta-analysis comparing the effect of all interventions on National Institutes of Health Stroke Scale (3 month follow-up) including SMD and 95% CI

Red and bold numbers are statistically significant. *HF-rTMS;* high-frequency repetitive transcranial magnetic stimulation, *LF-rTMS;* low-frequency repetitive transcranial magnetic stimulation, *BL-rTMS*; bilateral application of HF- rTMS and LF-rTMS, *iTBS*, intermittent theta-burst stimulation, *tDCS;* transcranial direct current stimulation

### AEs

AEs were reported in 29 studies (Table 9), including mild AEs (tingling, itching, burning, headache, fatigue, drowsiness, and dizziness), and severe AEs (stroke recurrence, worsened nerve injury indicated by changes in NIHSS or mRS scores, and epileptic seizure). Overall, no severe AEs were caused by stimulations in the included studies, and all mild AEs resolved spontaneously after treatment. The incidence rate of AEs for each protocol is shown in Table 10.

**Table 9:** Summary of adverse events

Study	Sessions	E/C	*n*	AE
Vink [27]	10	cTBS	28	headache(10 cases); Muscle pain(2 cases)
		sham	31	Nausea(1 case); headache(3 cases); Sensory impairment(1 case); Slowed thinking(2 cases)
Garrido [28]	7	dual-tDCS	29	itching(18) cases; tingling(10 cases); headache(3 cases); burning(3 cases); fatigued(2 cases)
		Sham	27	itching(14 cases); tingling(7 cases); headache(2 cases); burning(2 cases); fatigued(1 case)
Zhao [29]	12	dual-tDCS	30	slight tingling pain(1 case)
		Control	30	None
Komatsu [30]	10	HF-rTMS	22	None
		Control	22	None
Klomjai [19]	5	anodal-tDCS	20	tingling(50%); dizziness (20%); itching(10%); burning(10%); sleepiness(10%)
		cathodal-tDCS	18	
		dual-tDCS	20	
		Sham	20	None
Chen [16]	20	BL-rTMS	22	headache(1 case); tingling(1 case)
		Sham	22	None
Chen [31]	20	BL-rTMS	25	headache(3 cases); tingling(2 cases)
		HF-rTMS	25	
		LF-rTMS	25	
		Sham	25	None
Kim [32]	15	LF-rTMS	8	None
		Control	12	None
Ke [33]	10	HF-rTMS	32	None
		Sham	16	None
Bolognini [34]	10	dual-tDCS	16	No severe AEs
		Sham	16	No severe AEs
Du [20]	5	HF-rTMS	18	headache(2 cases)
		LF-rTMS	19	None
		Sham	16	None
Bornheim [36]	20	anodal-tDCS	23	tingling(22 cases); itching(15 cases); burning(9 cases); headache(2 cases)
		Sham	23	tingling(18 cases); itching(12 cases); burning(11 cases)
Watanabe [37]	10	iTBS	8	None
		LF-rTMS	7	None
		Sham	6	None
Long [38]	15	LF-rTMS	21	None
		BL-rTMS	21	None
		Sham	20	None
Li [39]	10	5 Hz rTMS	6	None
		Sham	6	None
Rabadi [40]	10	cathodal-tDCS	8	Tingling(NR)
		Sham	8	tingling(NR)
Oveisgharan [41]	10	dual-tDCS	10	None
		Sham	10	None
Guan [42]	10	5 Hz rTMS	13	None
		Sham	14	None
Guo [43]	10	HF-rTMS	7	None
		Sham	8	None
Du [44]	5	3 Hz rTMS	20	headache(3 cases); tingling(1 case)
		LF-rTMS	16	
		Sham	19	None
Matsuura [45]	5	LF-rTMS	10	None
		Sham	10	None
Sasaki [47]	5	HF-rTMS	31	None
		BL-rTMS	27	None
Kim [48]	5	LF-rTMS	22	None
		Sham	10	None
Sasaki [51]	5	LF-rTMS	11	None
		HF-rTMS	9	None
		Sham	9	None
Rossi [52]	5	anodal-tDCS	25	None
		Sham	25	None
Khedr [53]	5	3 Hz rTMS	16	None
		HF-rTMS	16	None
		Sham	16	None
Chang [54]	10	HF-rTMS	18	None
		Sham	10	None
Khedr [55]	5	LF-rTMS	12	None
		3 Hz rTMS	12	None
		Sham	12	None
Khedr [56]	10	3 Hz rTMS	26	headache(NR)
		Sham	26	None

**Table 10:** Incidence of adverse events

Treatment	LF-rTMS	HF-rTMS	BL-rTMS	cTBS	iTBS	dual-tDCS	anodal-tDCS	5 Hz-rTMS	3 Hz-rTMS	sham
No. of studies	8	8	3	1	1	3	2	2	2	19
Sample siza	110	153	70	28	8	69	48	19	28	317
treatment sessions	875	1160	890	280	80	663	585	190	140	2889
headache	0	2	1	10	0	3	2	0	0	5
Muscle pain	0	0	0	2	0	0	0	0	0	0
Nausea	0	0	0	0	0	0	0	0	0	1
Sensory impairment	0	0	0	0	0	0	0	0	0	1
Slowed thinking	0	0	0	0	0	0	0	0	0	2
itching	0	0	0	0	0	18	15	0	0	26
tingling	0	0	1	0	0	11	22	0	0	25
burning	0	0	0	0	0	3	9	0	0	13
fatigued	0	0	0	0	0	2	0	0	0	1
dizziness	0	0	0	0	0	0	0	0	0	0
sleepiness	0	0	0	0	0	0	0	0	0	0
Total probability	0.00%	0.17%	0.22%	4.29%	0.00%	5.58%	8.21%	0.00%	0.00%	2.56%

## Discussion

This NMA included 37 studies involving 1731 patients, and the differences in the efficacy between tDCS and rTMS protocols were compared, thereby providing a rationale for the recovery of motor function, ADL, and neurological function in early stroke patients following TMS and tDCS, as well as for the safety of early interventions. In terms of motor function, early BL-rTMS improved FMA-UE scores and achieved a long-term effect compared with other protocols; for the lower extremity motor function, none of the stimulation protocols included had a statistically significant difference in FMA-LE improvement from the control group. In terms of ADL, early BL-rTMS, LF-rTMS, HF-rTMS, 3 Hz-rTMS, iTBS, and cathodal-tDCS were all more effective than sham or conventional treatment alone, with LF-rTMS and BL-rTMS exerting long-term effects of improving mBI. Moreover, LF-rTMS, 3Hzr-TMS, HF-rTMS, BL-rTMS, and iTBS were more effective than sham in improving NIHSS scores, with BL-rTMS and LF-rTMS exerting long-term effects. To sum up, BL-rTMS is the optimal early stimulation protocol for ameliorating upper extremity motor function, ADL, and neurological function, achieving long-term effects. Besides, neither early rTMS nor tDCS led to severe AEs within 30 days after the onset of the disease.

Patients often experience multiple dysfunctions, primarily motor dysfunction, following a stroke, which can severely impact their quality of life [3]. FMA-UE and FMA-LE scales are designed to assess upper and lower extremity motor function, with good reliability and validity [57] In this NMA, early BL-rTMS was the most effective in improving upper extremity motor function in stroke patients. Stroke usually induces an imbalance of interhemispheric inhibition [58, 59], and BL-rTMS may contribute to motor functional improvement by restoring the balance of interhemispheric cortical excitability [60]. The effects of LF-rTMS, HF-rTMS, and BL-rTMS on cortical excitability in early stroke patients have been studied [16], and the results showed that both the amplitude of motor evoked potentials and resting motor thresholds are improved more significantly in the BL-rTMS group than other groups. Such improvement in motor function displays a significant correlation with the change in motor cortex excitability in the affected hemisphere [61]. BL-rTMS produces a synergistic effect by combining inhibitory and facilitatory stimulations [16, 62], enhancing the coordination of interhemispheric competition [20], which may account for the superiority of BL-rTMS.

In this NMA, LF-rTMS had the highest probability of being effective in improving the lower extremity motor function based on SUCRA, similar to previous findings [63]. An NMA on the effect of different types of rTMS on lower extremity motor function [63] has manifested that LF-rTMS ranks first in efficacy and only LF-rTMS outperforms sham in improving lower extremity motor function. In this NMA, however, the stimulations showed no statistically significant difference in the improvement of FMA-LE as compared to sham. The inconsistent findings may be attributed to the small number of studies involving FMA-LE and samples, and high heterogeneity. Therefore, it is recommended that more studies on the effect of early NIBS on FMA-LE should be included to further verify the efficacy of different stimulations on early stroke.

Post-stroke dysfunction seriously affects patients’ ADL. mBI is a widely-used tool for assessing ADL in stroke patients, with good reliability, validity, and sensitivity [64]. In this NMA, BL-rTMS was the best protocol for improving mBI after intervention and at 3-month follow-up, consistent with the findings of Chen et al. [65], who reported that BL-rTMS was the most effective in improving FMA, mBI, and NIHSS scores in acute stroke patients. Improvement in upper extremity motor function contributes to better ADL, the latter of which is not solely based on improvement in arm function on the hemiplegic side [66] but on a generalized treatment effect. Sasaki et al. [47] found that BL-rTMS is far better than HF-rTMS as assessed by the Brunnstrom Recovery Stage, possibly indicating the benefits of BL-rTMS in ameliorating the overall function in hemiplegic patients.

NIHSS scores are a good indicator of the severity of post-stroke nerve injury [67, 68]. In this NMA, BL-rTMS performed best in improving the NIHSS scores after early intervention and at 3-month follow-up. The reason is that with a synergistic effect of low- and high-frequency stimulation, BL-rTMS can excite/inhibit the corresponding cerebral cortex, thereby ameliorating the neurological function [16, 62].

In the acute stage (within one week after stroke onset), restorative therapy is initiated in relatively few studies [4, 7] NIBS is a promising therapy in the acute stage, but the safety of its early use, especially the risk of post-injury epileptic seizure [69], is one of the major concerns [70]. In this NMA, 21 out of the 37 included studies involved acute-phase interventions within one week in stroke (infarction and hemorrhage) by LF-rTMS, 3 Hz-rTMS, 5 Hz-rTMS, HF-rTMS, BL-rTMS, iTBS, cathodal-tDCS, anodal-tDCS, and dual-tDCS. No severe AEs were reported, while only mild AEs such as dizziness, tingling, fatigue, and itching occurred, which were relieved after resting, proving to a certain extent the safety of early intervention. Specifically, LF-rTMS, iTBS, and 5 Hz-rTMS showed a rate of AEs of 0%, exhibiting a better safety profile. Previous study [71] has concluded that LF-rTMS has higher safety; in particular, low-frequency stimulation helps to reduce cortical hyperexcitability in early stroke patients, thereby reducing the potential risk. However, not all of the included studies reported AEs in a strict and standardized manner, and thus further statistical analyses of the incidence of AEs following different interventions had to be abandoned. Therefore, it is recommended that AEs should be reported rigorously and in detail in future studies to provide guidance on the safety of early NIBS.

This study still had some limitations. First, the duration of intervention, site of stimulation, and severity of stroke varied across the included studies, leading to potential heterogeneity. Second, despite complete protocols of stimulation, the sample sizes were small in the iTBS and cTBS groups, each accounting for 2.53% of the total, which affected to some extent the quality of the findings. In addition, not all of the included studies reported AEs rigorously. Therefore, the safety of each intervention protocol remains to be further investigated. Finally, as non-English-language studies were not included in this NMA, relevant studies of other languages may be missed.

## Conclusion

BL-rTMS is the optimal stimulation protocol for improving upper extremity motor function, ADL, and neurological function in early stroke, with its positive effects persisting for at least three months. However, the conclusions need to be further verified. It is recommended that BL-rTMS should be used as an adjuvant therapy in early stroke rehabilitation in clinical practice. In the future, high-quality, large-sample, multicenter, long-term follow-up randomized controlled trials are required to validate these findings.

## Supplementary information

Below is the link to the electronic supplementary material.

ESM 1 (DOCX 561 KB)

## Data Availability

The data that support the findings of this study are available from the corresponding author upon reasonable request.

## Abbreviations

ADL: Activities of daily living
AEs: Adverse events
BL-rTMS: Bilateral application of high- and low-frequency repetitive transcranial magnetic stimulation
cTBS: Continuous theta-burst stimulation
FMA: Fugl-Meyer assessment scale
FMA-LE: Fugl-Meyer assessment scale for lower extremity
FMA-UE: Fugl-Meyer assessment scale for upper extremity
HF-rTMS: High-frequency repetitive transcranial magnetic stimulation
iTBS: Intermittent theta-burst stimulation
LF-rTMS: Low-frequency repetitive transcranial magnetic stimulation
mBI: modified Barthel index
NIBS: Non-invasive brain stimulation
NIHSS: National Institutes of Health Stroke Scale
NMA: Network meta-analysis
RCTs: Randomized controlled trials
RoB: Risk of bias
rTMS: Repetitive transcranial magnetic stimulation
tDCS: Transcranial direct current stimulation
TIA: Transient ischemia attack
