# Supplementary material for: Comparison of the efficacy of different protocols of repetitive transcranial magnetic stimulation and transcranial direct current stimulation on motor function, activities of daily living, and neurological function in patients with early stroke: a systematic review and network meta-analysis
Source: Neurol Sci. 2025 Feb 5;46(6):2479–98. doi: 10.1007/s10072-025-08000-5 (PMC12084249; doi:10.1007/s10072-025-08000-5)
Supplement: Supplementary file 1 — (DOCX 561 KB) [file 10072_2025_8000_MOESM1_ESM.docx]

**Supplementary Materials**

**NEUROLOGICAL SCIENCES**

**Comparison of the Efficacy of Different Protocols of Repetitive Transcranial Magnetic Stimulation and Transcranial Direct Current Stimulation on Motor Function, Activities of Daily Living, and Neurological Function in Patients with Early Stroke: A Systematic Review and Network Meta-analysis**

Xueyi Ni, Zinan Yuan, Ruimou Xie, Xiaoxue Zhai, Xiang Cheng, Yu Pan*

Department of Rehabilitation Medicine, Beijing Tsinghua Changgung Hospital, Beijing 102218, China

***Corresponding author:** Yu Pan

Email: panyu@btch.edu.cn

Tel: +86 56119272

Address: Beijing Tsinghua Changgung Hospital, Tsinghua University, No. 168, Litang Road, Changping District, Beijing, China

**Supplementary File 1** Search Strategies

**PubMed**

(((((((((((((Stroke*[Title/Abstract]) OR (Cerebrovascular Accident*[Title/Abstract])) OR (Cerebrovascular Apoplexy[Title/Abstract])) OR (Brain Vascular Accident*[Title/Abstract])) OR (Cerebrovascular Stroke*[Title/Abstract])) OR (Apoplexy[Title/Abstract])) OR (Cerebral Stroke*[Title/Abstract])) OR (Cerebral infarction[Title/Abstract])) OR (Intracerebral hemorrhage[Title/Abstract])) OR (Infarction[Title/Abstract])) OR ("Stroke"[Mesh])) AND (((((((((((((non-invasive brain stimulation*[Title/Abstract]) OR (Transcranial Direct Current Stimulation*[Title/Abstract])) OR (tDCS*[Title/Abstract])) OR (Cathodal Stimulation Transcranial Direct Current Stimulation[Title/Abstract])) OR (Cathodal Stimulation tDCS*[Title/Abstract])) OR (Transcranial Electrical Stimulation*[Title/Abstract])) OR (Anodal Stimulation Transcranial Direct Current Stimulation[Title/Abstract])) OR (Anodal Stimulation tDCS*[Title/Abstract])) OR (Repetitive Transcranial Electrical Stimulation*[Title/Abstract])) OR (Transcranial Magnetic Stimulation*[Title/Abstract])) OR (repetitive transcranial magnetic stimulation*[Title/Abstract])) OR (rTMS[Title/Abstract])) OR (("Transcranial Direct Current Stimulation"[Mesh]) OR "Transcranial Magnetic Stimulation"[Mesh]))) AND ((((((Acute[Title/Abstract]) OR (early[Title/Abstract])) OR ("early phase"[Title/Abstract])) OR ("early stage"[Title/Abstract])) OR ("early period"[Title/Abstract])) OR ("Recent Stroke"[Title/Abstract]))) AND (randomized controlled trial[Publication Type] OR randomized[Title/Abstract] OR placebo[Title/Abstract])

Date：7 October 2023

Result：171

**Web of Science**

1：(((((((((TS=(Stroke*)) OR TS=(Cerebrovascular Accident*)) OR TS=(Cerebrovascular Apoplexy)) OR TS=(Brain Vascular Accident*)) OR TS=(Cerebrovascular Stroke*)) OR TS=(Apoplexy)) OR TS=(Cerebral Stroke*)) OR TS=(Cerebral infarction)) OR TS=(Intracerebral hemorrhage)) OR TS=(Infarction)

2：(((((((((((((TS=("Transcranial Direct Current Stimulation")) OR TS=("Transcranial Magnetic Stimulation")) OR TS=("non-invasive brain stimulation*")) OR TS=("Transcranial Direct Current Stimulation*")) OR TS=(tDCS)) OR TS=("Cathodal Stimulation Transcranial Direct Current Stimulation")) OR TS=("Cathodal Stimulation")) OR TS=("Transcranial Electrical Stimulation*")) OR TS=("Anodal Stimulation Transcranial Direct Current Stimulation")) OR TS=("Anodal Stimulation")) OR TS=("Repetitive Transcranial Electrical Stimulation*")) OR TS=("Transcranial Magnetic Stimulation*")) OR TS=("repetitive transcranial magnetic stimulation*")) OR TS=("rTMS")

3：(((((TS=(Acute)) OR TS=(early)) OR TS=("early phase")) OR TS=("early stage")) OR TS=("early period")) OR TS=("Recent Stroke ")

4：((((TS=("randomized controlled trial")) OR TS=(randomized)) OR TS=(placebo)) OR TS=(controlled)) OR TS=(blind)

5：#1 AND #4 AND #3 AND #4

Date：7 October 2023

Result：438

**Embase**

#1：'cerebrovascular accident'/exp

#2：stroke* OR 'cerebrovascular accident*' OR 'cerebrovascular apoplexy' OR 'brain vascular accident*' OR 'cerebral stroke*' OR 'cerebral infarction' OR 'intracerebral hemorrhage' OR infarction

#3：#1 OR #2

#4：'transcranial direct current stimulation'/exp

#5：'transcranial magnetic stimulation'/exp

#6：'non-invasive brain stimulation*' OR 'transcranial direct current stimulation*' OR tdcs* OR 'transcranial electrical stimulation*' OR 'repetitive transcranial electrical stimulation*' OR 'transcranial magnetic stimulation*' OR 'repetitive transcranial magnetic stimulation*' OR 'rtms' OR 'continuous theta-burst stimulation' OR 'ctbs' OR 'intermittent theta-burst stimulation' OR 'itbs'

#7：#4 OR #5 OR #6

#8：acute OR early OR 'early phase' OR 'early stage' OR 'early period' OR 'recent stroke'

#9：'randomized controlled trial'/exp

#10：randomized OR placebo OR controlled OR blind

#11：#9 OR #10

#12：#3 AND #7 AND #8 AND #11

Date：7 October 2023

Result：451

**Cochrane Library**

#1：MeSH descriptor: [Stroke] explode all trees

#2：(stroke* OR 'cerebrovascular accident*' OR 'cerebrovascular apoplexy' OR 'brain vascular accident*' OR 'cerebral stroke*' OR 'cerebral infarction' OR 'intracerebral hemorrhage' OR infarction):ti,ab,kw

#3：#1 OR #2

#4：MeSH descriptor: [Transcranial Direct Current Stimulation] explode all trees

#5：MeSH descriptor: [Transcranial Magnetic Stimulation] explode all trees

#6：('non-invasive brain stimulation*' OR 'transcranial direct current stimulation*' OR tdcs* OR 'transcranial electrical stimulation*' OR 'repetitive transcranial electrical stimulation*' OR 'transcranial magnetic stimulation*' OR 'repetitive transcranial magnetic stimulation*' OR 'rtms' OR 'continuous theta-burst stimulation' OR 'ctbs' OR 'intermittent theta-burst stimulation' OR 'itbs') :ti,ab,kw

#7：#4 OR #5 OR #6

#8：(acute OR early OR 'early phase' OR 'early stage' OR 'early period' OR 'recent stroke') :ti,ab,kw

#9：MeSH descriptor: [Randomized Controlled Trial] explode all trees

#10：(randomized OR placebo OR controlled OR blind) :ti,ab,kw

#11：#9 OR #10

#12：#3 AND #7 AND #8 AND #11

Date：7 October 2023

Result：411

**Supplementary File 2** The convergence diagnostic diagram

**Abbreviations:** HF-rTMS, high-frequency repetitive transcranial magnetic stimulation; LF-rTMS, low-frequency repetitive transcranial magnetic stimulation; BL-rTMS, bilateral application of HF- rTMS and LF-rTMS; iTBS, intermittent theta-burst stimulation; cTBS, continuous theta-burst stimulation; tDCS, transcranial direct current stimulation


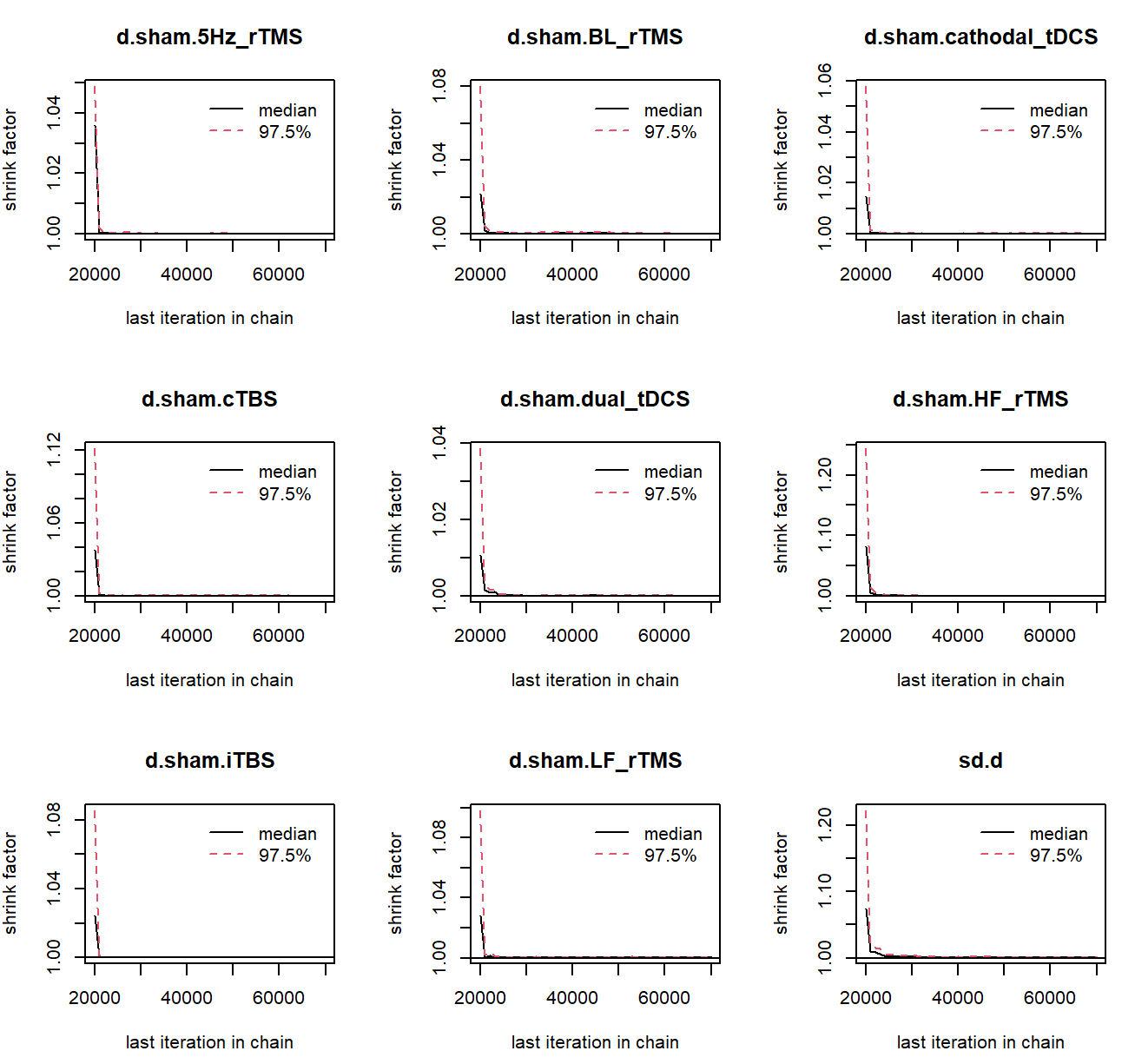


**2(A): Fugl-Meyer assessment scale for upper extremity (end of treatment)**


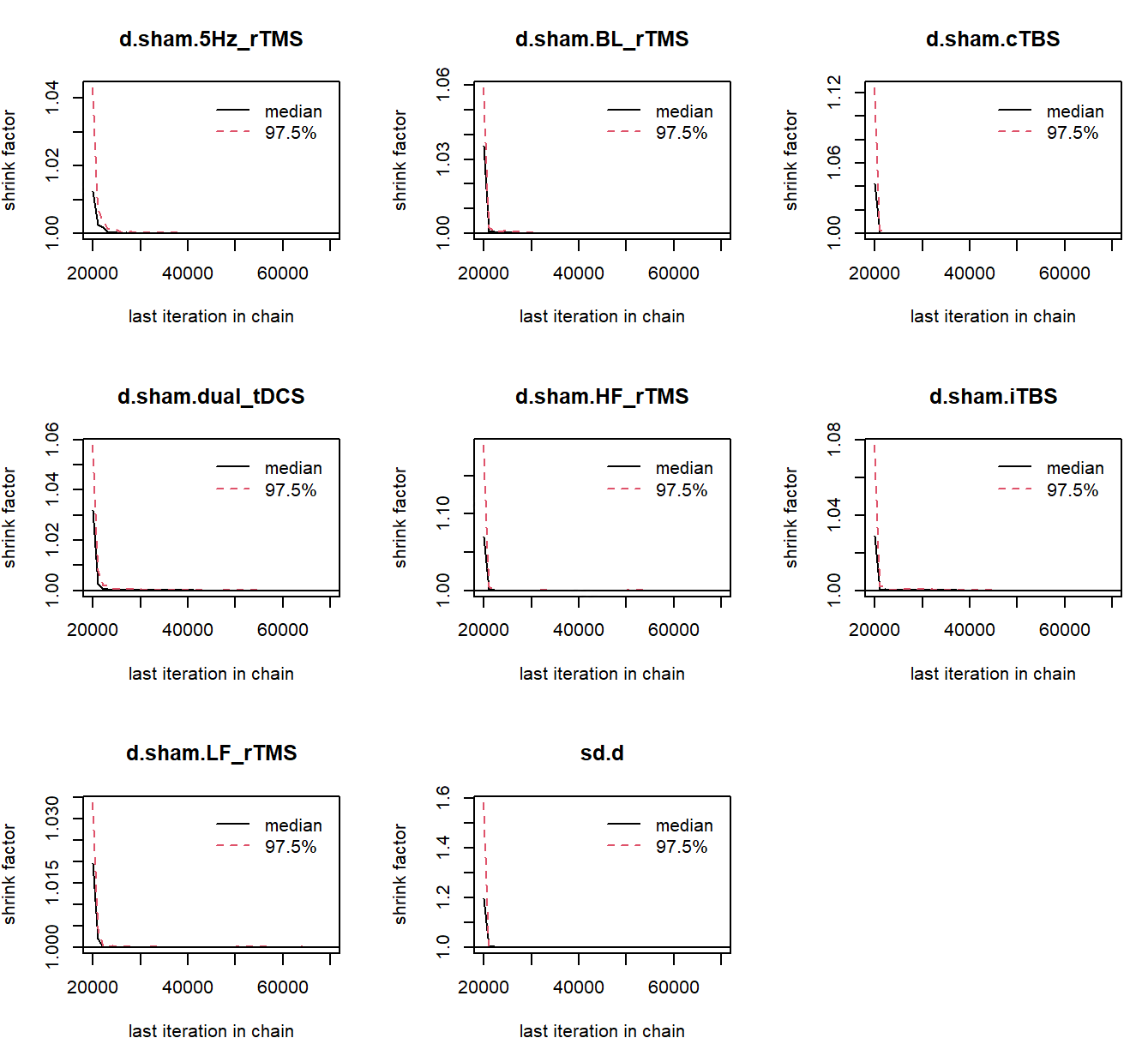


**2 (B): Fugl-Meyer assessment scale for upper extremity (3 month follw-up)**


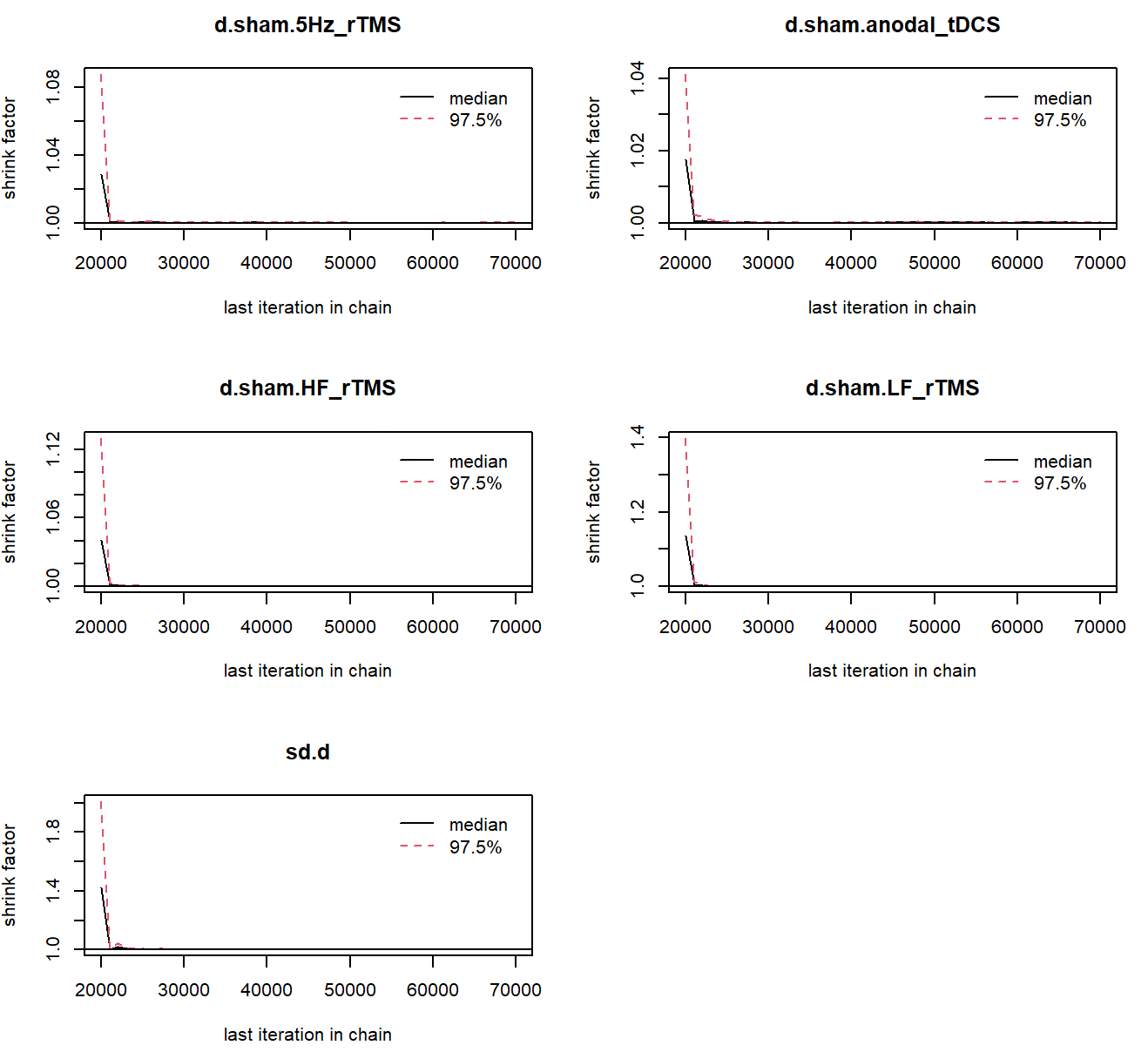


**2(C): Fugl-Meyer assessment scale for lower extremity (end of treatment)**


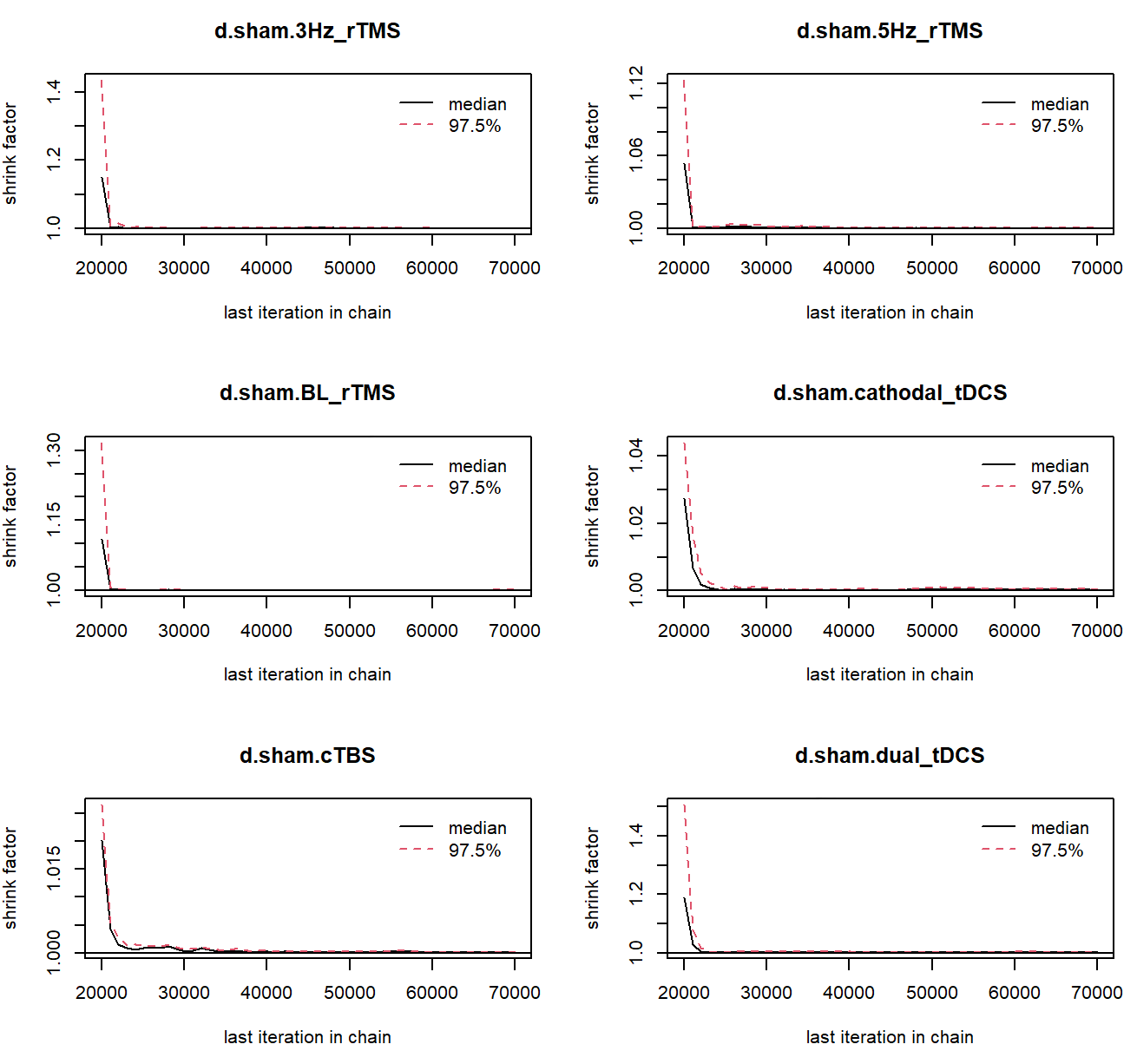


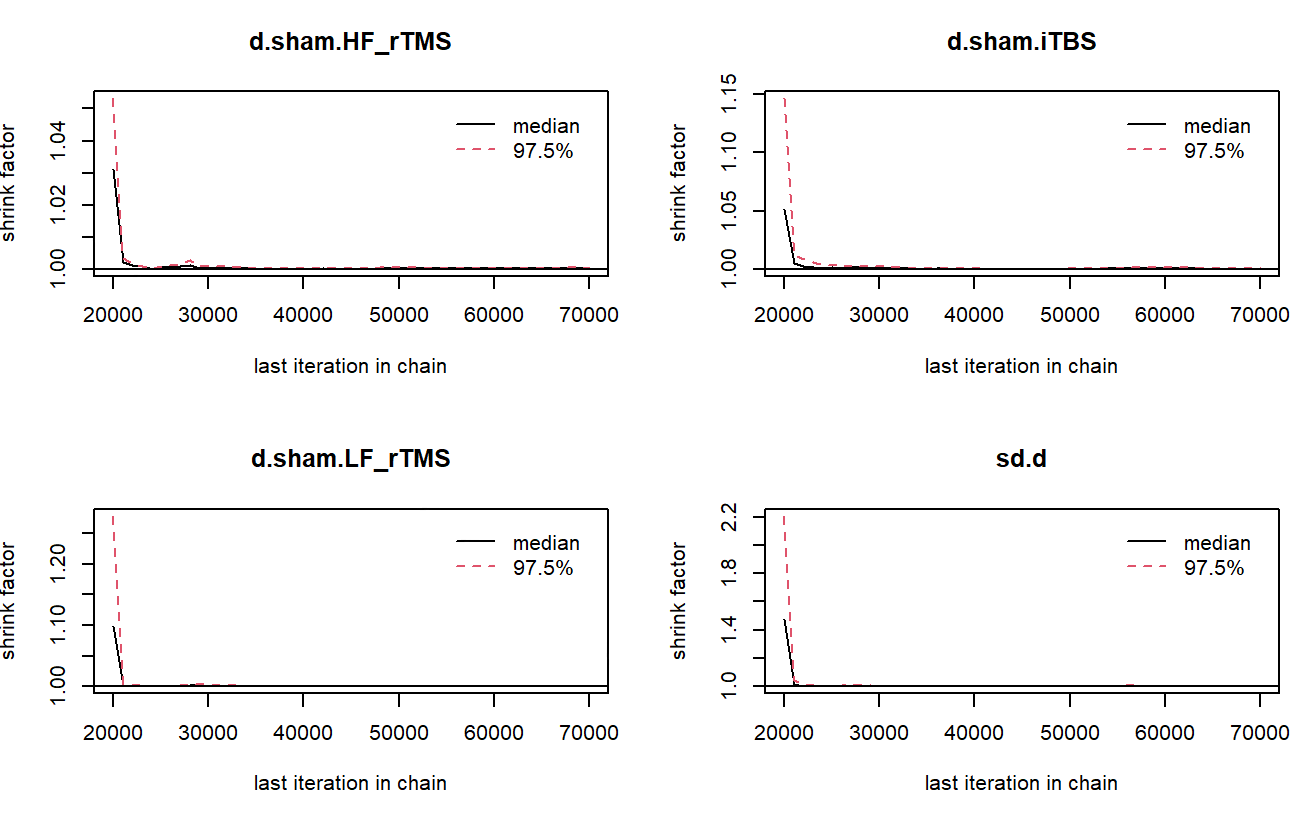


**2 (D): modified Barthel Index (end of treatment)**


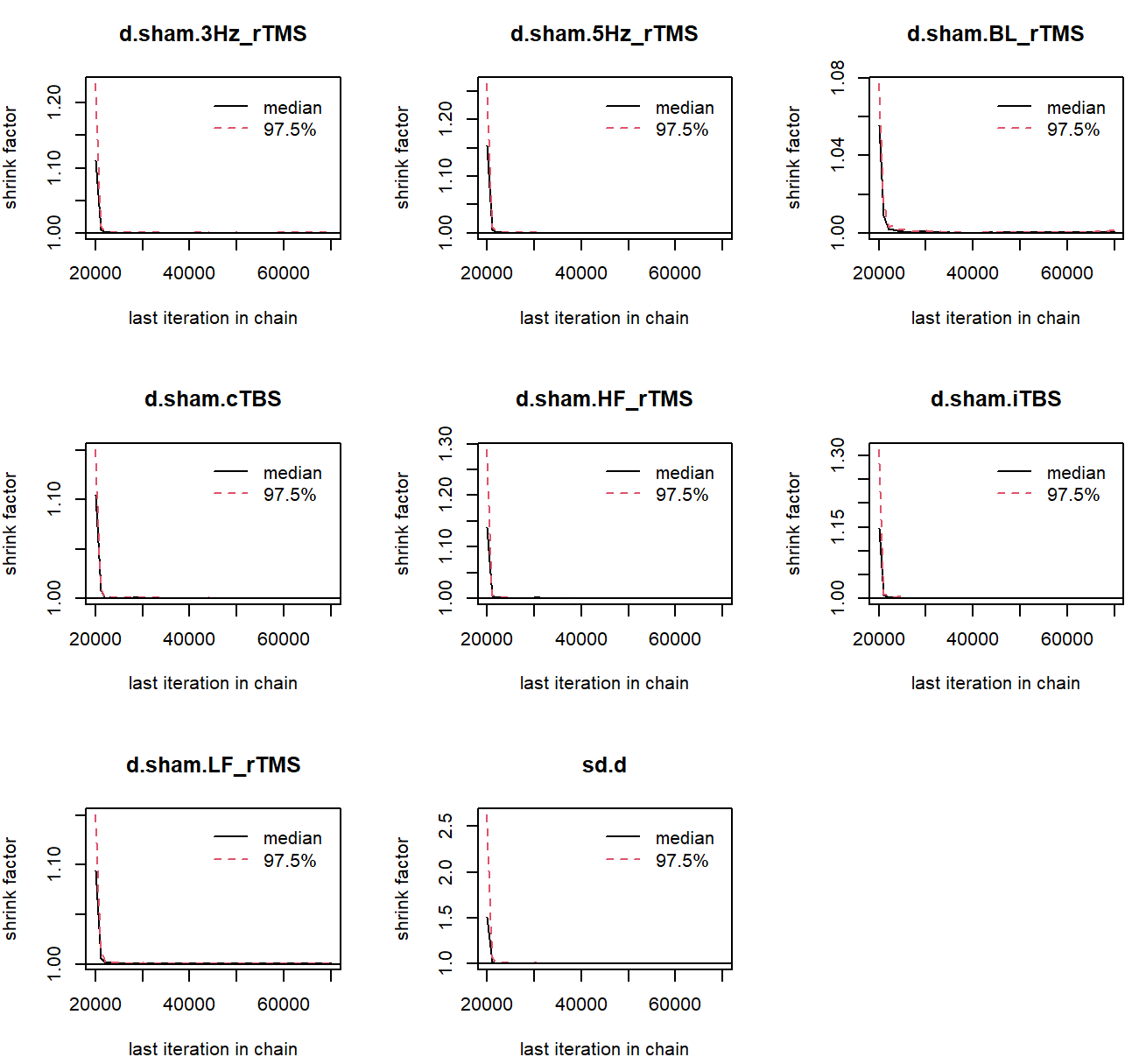


**2 (E): modified Barthel Index (3 month follw-up)**


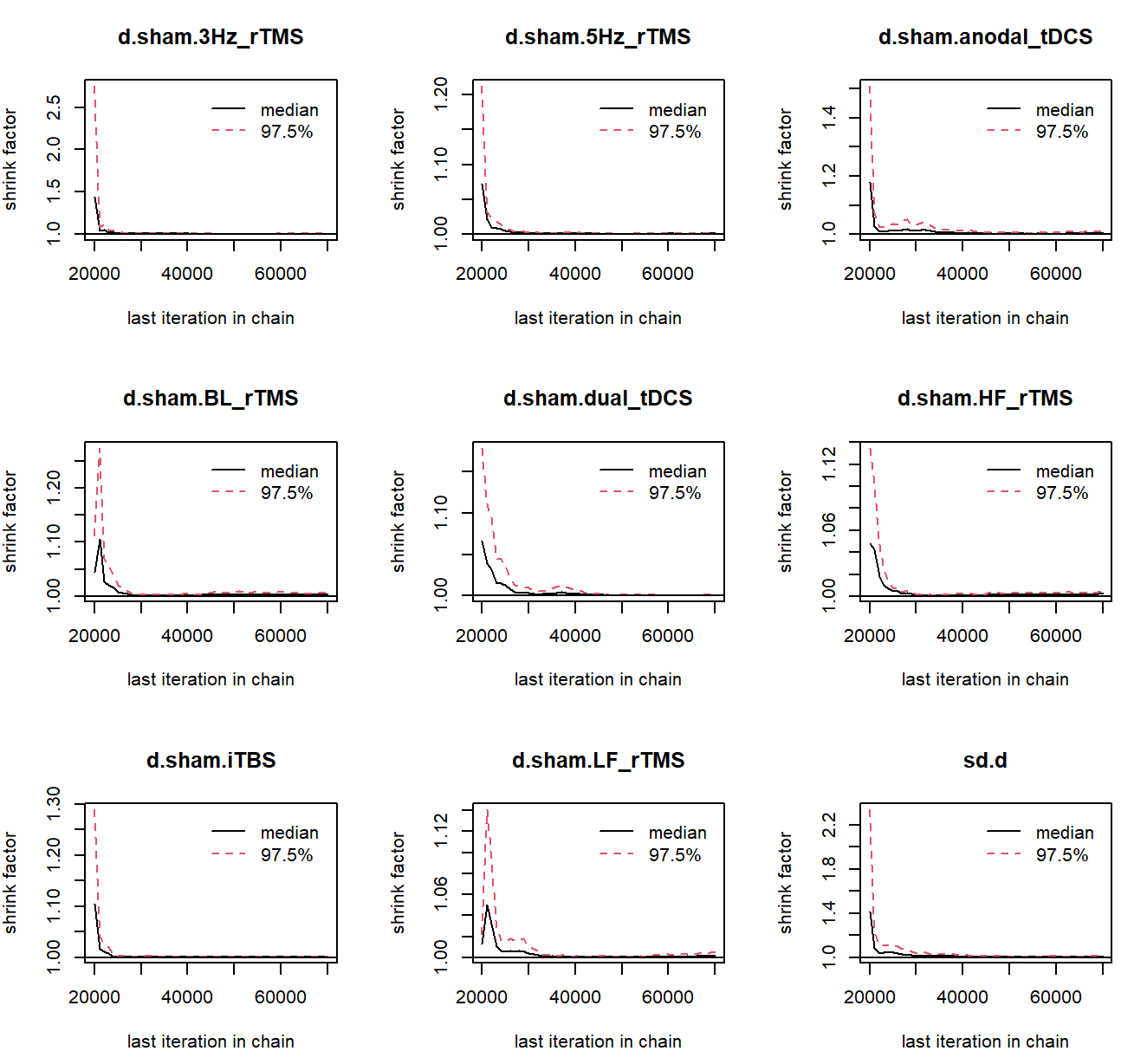


**2 (F): National Institute of Health Stroke Scale (end of treatment)**


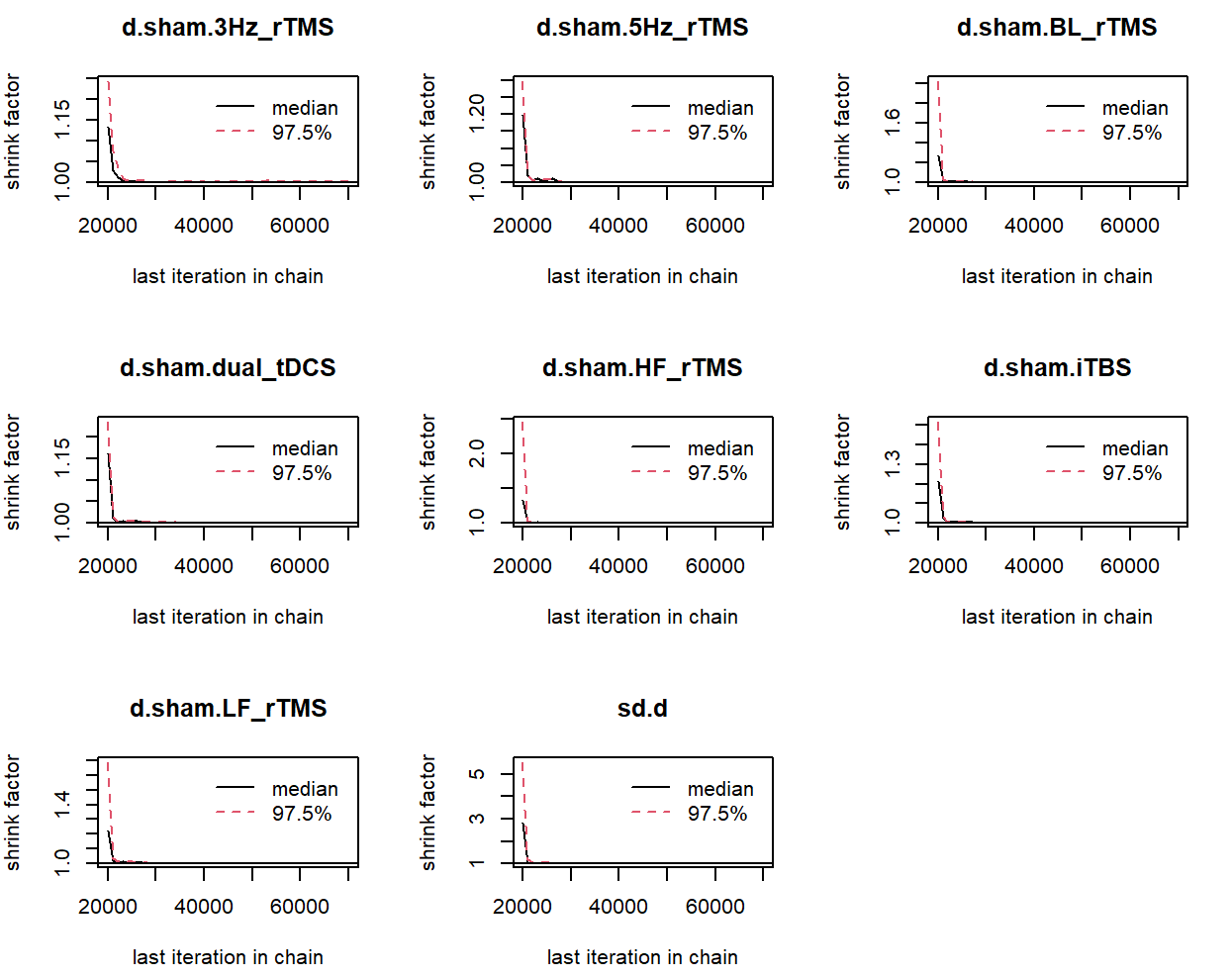


**2(G): National Institute of Health Stroke Scale (3 month follw-up)**
